# *Plasmodium* subtilisin-like protease 1 (SUB1): Insights into the active-site structure, specificity and function of a pan-malaria drug target

**DOI:** 10.1016/j.ijpara.2012.04.005

**Published:** 2012-05-15

**Authors:** Chrislaine Withers-Martinez, Catherine Suarez, Simone Fulle, Samir Kher, Maria Penzo, Jean-Paul Ebejer, Kostas Koussis, Fiona Hackett, Aigars Jirgensons, Paul Finn, Michael J. Blackman

**Affiliations:** a Division of Parasitology, MRC National Institute for Medical Research (NIMR), Mill Hill, London NW7 1AA, UK; b InhibOx Ltd., New Road, Oxford OX1 1BY, UK; c Latvian Institute of Organic Synthesis, Aizkraukles 21, LV-1006 Riga, Latvia

**Keywords:** *Plasmodium*, SUB1, Subtilisin, Substrate specificity, Alpha-ketoamide

## Abstract

Release of the malaria merozoite from its host erythrocyte (egress) and invasion of a fresh cell are crucial steps in the life cycle of the malaria pathogen. Subtilisin-like protease 1 (SUB1) is a parasite serine protease implicated in both processes. In the most dangerous human malarial species, *Plasmodium falciparum*, SUB1 has previously been shown to have several parasite-derived substrates, proteolytic cleavage of which is important both for egress and maturation of the merozoite surface to enable invasion. Here we have used molecular modelling, existing knowledge of SUB1 substrates, and recombinant expression and characterisation of additional *Plasmodium* SUB1 orthologues, to examine the active site architecture and substrate specificity of *P. falciparum* SUB1 and its orthologues from the two other major human malaria pathogens *Plasmodium vivax* and *Plasmodium knowlesi*, as well as from the rodent malaria species, *Plasmodium berghei*. Our results reveal a number of unusual features of the SUB1 substrate binding cleft, including a requirement to interact with both prime and non-prime side residues of the substrate recognition motif. Cleavage of conserved parasite substrates is mediated by SUB1 in all parasite species examined, and the importance of this is supported by evidence for species-specific co-evolution of protease and substrates. Two peptidyl alpha-ketoamides based on an authentic PfSUB1 substrate inhibit all SUB1 orthologues examined, with inhibitory potency enhanced by the presence of a carboxyl moiety designed to introduce prime side interactions with the protease. Our findings demonstrate that it should be possible to develop ‘pan-reactive’ drug-like compounds that inhibit SUB1 in all three major human malaria pathogens, enabling production of broad-spectrum antimalarial drugs targeting SUB1.

## Graphical abstract

**Graphical abstract:**

**Figure:**
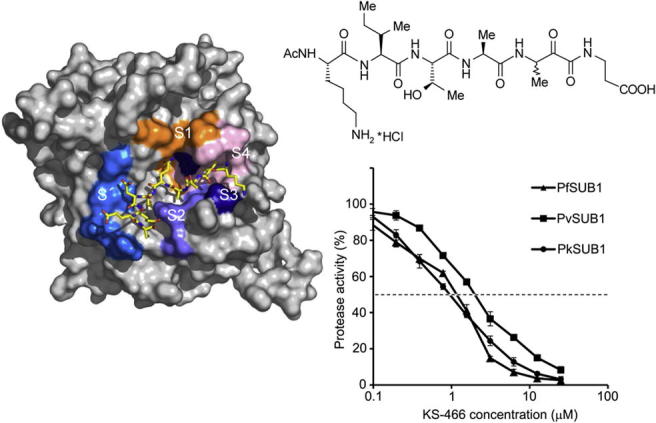

**Highlights:**

► Molecular modelling of subtilisin-like protease 1 (SUB1) of three human malaria pathogens shows similarity in active site. ► Experimental examination of *Plasmodium falciparum* (Pf)SUB1 demonstrates unusual features of the active site. ► Recombinant expression of active *Plasmodium vivax* (Pv)SUB1, *Plasmodium knowlesi* (Pk)SUB1 and *Plasmodium berghei* (Pb)SUB1. ► Evidence for co-evolution of SUB1 orthologues and substrates following speciation. ► Production of substrate-based inhibitors with broad activity against SUB1 from three major human malarial pathogens.

## 1 Introduction

The continuing appearance and spread of drug-resistant malaria – in particular alarming reports of resistance to artemisinin-based drugs (recently reviewed by O’Brien et al., 2011) – emphasises the need to better understand the biology of the malaria parasite and to continuously explore new routes to antimalarial drug discovery. Of the five *Plasmodium* spp. known to infect humans, *Plasmodium falciparum* is the most virulent and causes by far the greatest mortality. However, *Plasmodium vivax* is also responsible for extensive morbidity and can cause severe disease (Price et al., 2007, 2009), whilst *Plasmodium knowlesi* has emerged as an agent of potentially fatal malaria in parts of Malaysia (e.g. William et al., 2011). Work over the last two decades has demonstrated that proteolytic enzymes play crucial roles in the malarial life cycle. In the asexual blood-stages, responsible for all of the clinical manifestations of malaria, rupture of schizonts to allow egress of invasive merozoites can be efficiently blocked by the cysteine protease inhibitor E64 (Salmon et al., 2001; Wickham et al., 2003; Soni et al., 2005; Boyle et al., 2010), implicating at least one cysteine protease. Invasion of erythrocytes by released merozoites, on the other hand, which involves merozoite surface proteins as well as proteins discharged from secretory organelles called micronemes and rhoptries, is insensitive to E64 (Boyle et al., 2010) but can be prevented by serine protease inhibitors (Hadley et al., 1983). Work to identify the enzyme(s) involved in egress and invasion revealed that the *Plasmodium* genome encodes three proteins belonging to the subtilase superfamily (clan SB, according to MEROPS nomenclature) (Rawlings et al., 2012), a group of usually secreted serine proteases that play diverse roles across evolution. Named SUB1, SUB2 and SUB3, single-copy orthologues of all three are evident in all *Plasmodium* genomes examined, including those of *P. knowlesi* and *P. vivax*. Gene disruption experiments in *P. falciparum* have shown that SUB3 is not required for asexual blood-stage growth in vitro (O’Donnell and Blackman, unpublished data). By contrast, SUB2 acts as an essential membrane-bound ‘sheddase’ which cleaves proteins from the surface of the invading merozoite (Harris et al., 2005). SUB1 is the best studied of the three subtilases, and previous work from this laboratory has shown that *P. falciparum* SUB1 (PfSUB1: PlasmodDB ID PFE0370c, MEROPS ID S08.012) is expressed late in schizont maturation, accumulating in sub-cellular organelles of the developing merozoites termed exonemes (Yeoh et al., 2007). Just prior to egress, PfSUB1 is released into the lumen of the parasitophorous vacuole (PV), where it proteolytically cleaves several important parasite proteins. These include members of a family of papain-like proteins called the serine rich antigen (SERA) family, and the merozoite surface proteins, MSP1, MSP6 and MSP7 (Yeoh et al., 2007; Koussis et al., 2009). The function of these processing events is unknown. However, MSP1 is essential (Combe et al., 2009), and mutations that interfere with its processing are deleterious to parasite growth (Child et al., 2010). There is also strong evidence that several of the SERA family members play essential roles (McCoubrie et al., 2007; Putrianti et al., 2010), and it has been suggested that processing of the SERA proteins may convert them to active proteases (Blackman, 2008). PfSUB1 appears to be indispensable in the asexual blood-stage *P. falciparum* life cycle, and the finding that selective pharmacological inhibition of PfSUB1 blocks egress and reduces the invasiveness of released merozoites shows that PfSUB1 is a ‘druggable’ enzyme (Yeoh et al., 2007; Arastu-Kapur et al., 2008; Koussis et al., 2009). Maturation of PfSUB1 appears to be dependent on the activity of parasite dipeptidyl aminopeptidase 3 (DPAP3), a papain-like cysteine protease (Arastu-Kapur et al., 2008), and this may be one of the proteases targeted by E64 in the egress-inhibition experiments referred to above.

Subtilases are unified by the characteristic αβ-fold of their single lobe catalytic domain and the order of the ‘catalytic triad’ residues in their primary sequence. These residues differ between the two major families of the clan, being Glu-Asp-Ser in the case of the S53 (sedolisin) family, and Asp-His-Ser in the case of the S8 family to which the *Plasmodium* SUB proteases belong (Wlodawer et al., 2003). Further subdivisions of the S8 family have been made based on sequence homologies, and the *Plasmodium* sequences show most similarity to members of the subtilisin subfamily S8A of predominantly bacterial enzymes as defined by Siezen and Leunissen (1997). Subtilisins are generally expressed as zymogens that undergo activation in one or more (usually autocatalytic) processing steps, releasing a prodomain (PD) to liberate an enzymatically active catalytic domain. The substantial homology between the primary sequence of the PfSUB1 catalytic domain and that of several bacterial subtilisins, the structures of which have been determined by X-ray crystallography, allowed us in earlier work to build a homology model of the PfSUB1 catalytic domain (Withers-Martinez et al., 2002). This showed that all of the important structural features characteristic of subtilases are present in PfSUB1. These include the eight major α-helix and 11 β-strand elements that comprise the structurally conserved core of subtilase catalytic domains, the presence of at least three potential calcium-binding sites, and three predicted intramolecular disulphide bonds. The substrate binding site within subtilisin catalytic domains forms a prominent surface cleft or groove that allows extended interactions with at least six substrate residues flanking the scissile bond (P4 to P2′ according to the nomenclature of Schechter and Berger, 1967). The most important of these interactions are generally within the S4 pocket, a particularly deep indentation within the binding groove, and the S1 pocket which is only slightly less deep. Our initial analysis of the substrate binding site within the PfSUB1 homology model (Withers-Martinez et al., 2002) was made in the light of the only substrate sequence known at that time, the _213_LVSAD ↓ NIDIS_222_ motif (scissile bond indicated by a downward-pointing arrow, numbering based on PlasmodDB ID PFE0370c) within PfSUB1 itself at which autocatalytic cleavage takes place during protease maturation (Sajid et al., 2000). The overall hydrophobic character of the predicted PfSUB1 S4 pocket and the contrasting polar nature of its S1 pocket was in accord with the substrate sequence, as these pockets accommodate, respectively, the aliphatic P4 Val and hydrophilic P1 Asp residues. The importance of these sub-site interactions was experimentally addressed using several derivatives of an *N*-acetylated peptide substrate, Ac-LVSADNIDIS, revealing that although some flexibility is tolerated at the P1 position, PfSUB1 cannot cleave substrates containing a P1 Leu residue, whilst replacement of the P4 Val with Ala inhibits cleavage and replacement with Lys completely ablates cleavage. The results of this initial analysis, together with peptide alanine scanning studies and the subsequent mapping of cleavage sites in a number of authentic endogenous protein substrates of PfSUB1 (Yeoh et al., 2007; Koussis et al., 2009), enabled us to assemble a consensus PfSUB1 recognition motif of Ile/Leu/Val/Thr-Xaa-Gly/Ala-Paa(not Leu) ↓ Xaa (where Xaa is any amino acid residue and Paa tends to be a polar residue). We also noticed a striking tendency for residues with acidic (Glu, Asp) or hydroxyl-containing (Ser, Thr) side-chains at one or more of the proximal five prime side positions in authentic target sequences, an unexplained and intriguing observation since no previously-studied S8A family subtilisin demonstrates strong prime side substrate preference. Recent use of the consensus motif to perform bioinformatic searches of the predicted *P. falciparum* proteome for new PfSUB1 substrates proved productive, enabling firm identification of the parasite proteins RAP1 and MSRP2 as additional substrates (Silmon de Monerri et al., 2011). However, the large number of false positives arising from that screen demonstrated that our current understanding of PfSUB1 specificity is incomplete.

An enhanced understanding of the structural elements important for recognition of target sequences would improve algorithms for prediction of biologically relevant PfSUB1 substrates – essential for a complete understanding of the function of the enzyme – and would aid directed design of selective inhibitors. In addition, characterisation of SUB1 orthologues from other *Plasmodium* spp. of clinical and/or experimental importance would provide valuable information on conserved functions as well as on the wider potential of SUB1 as a drug target. Towards these aims, we report here a structural investigation of the architecture of the PfSUB1 active site cleft based on modelling studies. Homology models of PfSUB1 bound to different target sequences now known to be cleaved in trans in authentic physiological substrates reveal structural features required for binding and provide a rational interpretation of the substrate specificity found in our previous experiments. These results are corroborated by molecular dynamics (MD) simulations, which provide further insights into the structural determinants for substrate binding. By comparing the PfSUB1 active site with that of the *P. vivax* and *P. knowlesi* orthologues, as well that of the widely used experimental rodent malaria model, *Plasmodium berghei*, we identify unusual features of the SUB1 active site that may be exploited in the design of SUB1 inhibitors. We show that there are no substantial differences between the active sites of the SUB1 orthologues from the human pathogens, whereas the *P. berghei* orthologue displays evidence of diversification in specificity and co-evolution of the enzyme and its substrates. We isolated a ‘core’ active domain of recombinant PfSUB1 (rPfSUB1) and describe the recombinant expression and partial characterisation of the other SUB1 orthologues under investigation. Finally, we used the recombinant enzymes to validate some major predictions from our modelling work, and to present a proof-of-principle that substrate-based inhibitors can be developed that target SUB1 orthologues from all three major human malaria pathogens.

## 2 Materials and methods

### 2.1 Expression and purification of recombinant proteins for PfSUB1, P. vivax SUB1 (PvSUB1), P. knowlesi SUB1 (PkSUB1) and P. berghei SUB1 (PbSUB1)

Recombinant expression and purification of PvSUB1, PkSUB1 and PbSUB1 (PlasmoDB ID PVX_097935, PKH_102540 and PBANKA_110710, respectively) used a protocol adapted from that previously described for rPfSUB1 (Withers-Martinez et al., 2002). Briefly, recodonised synthetic genes based on codon usage of Sf9 insect cells and encoding C-terminal six histidine (6-His) tags were produced by GeneArt (Invitrogen, UK), sub-cloned into the pFastBac1 expression vector (Invitrogen), then used to produce recombinant baculovirus using the Bac-to-Bac system (Invitrogen) according to the manufacturer’s instructions. Large scale protein production was achieved by infecting 100 ml of Tn5 cells at 2 × 10^6^ cells/ml in Sf900 II protein-free medium (Invitrogen) containing 0.5 μg/ml of tunicamycin, at a multiplicity of infection of 1–2 in a 500 ml Erlenmeyer flask, and swirling the cells at 85 rpm for 3–4 days at 27 °C. Approximately 3 L of accumulated culture supernatant were clarified, then the secreted recombinant protein purified in a two-step procedure using Blue Sepharose CL-6B (Sigma, UK) and high performance Ni–NTA agarose resin (Qiagen, UK), as described previously (Withers-Martinez et al., 2002) except that all elution buffers contained 10 mM 3-[(3cholamidopropyl)-dimethylammonio] propanesulfonate CHAPS and the Ni–NTA elution buffer contained 250 mM imidazole. If not to be processed further with chymotrypsin, purified proteins were concentrated and buffer-exchanged into 150 mM NaCl, 20 mM Tris–HCl pH 8.2, using a 10 kDa cut-off Centricon-70 (Millipore, UK). Protein yields varied from 0.38 mg/L of insect cell culture for rPfSUB1 to ∼4 mg/L for rPvSUB1.

### 2.2 Chymotrypsin digestion of recombinant SUB1

Purified rPfSUB1 in Ni–NTA elution buffer (150 mM NaCl, 20 mM Tris–HCl, pH 8.2, 10 mM CHAPS, 250 mM imidazole) was supplemented with bovine pancreatic α-chymotrypsin (Sigma catalogue No. C3142, type VII, TLCK-treated) to a final concentration of 5 μg/ml (∼0.2 U/ml) and incubated at 25 °C for 3–4 h. The chymotrypsin was inactivated by addition of phenylmethylsulfonyl fluoride (PMSF) to 2 mM from a 100 mM stock in isopropanol (note that PfSUB1 is unaffected by PMSF; Withers-Martinez et al., 2002). The digested protein was finally purified by chromatography on a HiLoad 26/60 Superdex 200 prep-grade column (GE Healthcare, UK) equilibrated in 20 mM Tris–HCl, 150 mM NaCl, pH 8.2. N-terminal sequencing by Edman degradation of the chymotrypsin-digested rPfSUB1 and other proteins was performed at the Protein and Nucleic Acid Chemistry Facility (University of Cambridge, UK). Electrospray mass spectrometry was used to establish the molecular mass of the chymotrypsin-digested rPfSUB1 as described previously (Howell et al., 2003). An identical process was used for chymotrypsin-treatment of rPvSUB1, rPkSUB1 and rPbSUB1.

### 2.3 Protease activity and specificity assays

Unlabelled *N*-acetylated synthetic peptides and fluorogenic substrate Ac-KITAQ-AMC were purchased from BIOMATIK (USA) or Pepceuticals (UK). All other fluorogenic peptide substrates used in this study were produced in-house by labelling both cysteine side-chains of the following peptides with 6-iodoacetamido tetramethylrhodamine (6-IATR): Ac-CKITAQDDEESC (SERA4st1F-6R12); Ac-CITAQDDEEC (called SERA4st1F-6R); and Ac-CITAQSDEEC (SERA4st1F-6R-S). Labelling, purification and use of these peptides was as described previously (Blackman et al., 2002; Withers-Martinez et al., 2002; Yeoh et al., 2007). The labelled peptides exhibit low fluorescence due to non-covalent, concentration-dependent dimerization of the rhodamines. Cleavage anywhere within the peptide backbone allows dissociation of the rhodamine dimer and consequent fluorescence increase. One unit (1 U) of rPfSUB1 is defined as the amount of protease that hydrolyses 1 pmol of SERA4st1F-6R12 in 1 min at a substrate concentration of 0.1 μM in digestion buffer (25 mM Tris–HCl, pH 8.2, 12 mM CaCl_2_, 25 mM CHAPS) at 21 °C. For kinetic assays, fluorogenic substrate solutions (0.1 μM in digestion buffer) were supplemented with purified recombinant protease (usually ∼1 U/ml), and fluorescence increase continuously monitored with time at 21 °C as described previously (Blackman et al., 2002) using a Cary Eclipse fluorescence spectrophotometer (Varian, UK) equipped with a 96-well microplate reader accessory. Progress curves are displayed as individual reads from a single well of a kinetic assay; readings taken from triplicate wells in the same experiments showed no more than 2% variation from the plots shown at individual time-points. For determination of IC50 (half maximal inhibitory concentration) values for peptidyl ketoamides KS-182 and KS-466, wells were supplemented in triplicate with various concentrations of the test compounds, diluted 1:100 from stock serial dilutions in DMSO, prior to addition of substrate. Initial hydrolysis rates were then plotted against inhibitor concentration. Determination of apparent tight binding inhibition constant *K_i_*_(app)_ values was performed as described previously (Jean et al., 2003) using the method of Nicklin and Barrett (1984), which uses the slope of the progress curve in the absence of the inhibitor under test (the initial velocity *v*_o_), and the slope of the curve immediately following addition of inhibitor (the inhibition velocity *v_i_*). A plot of (*v*_o_/*v_i_*) − 1 versus inhibitor concentration has a slope of 1/*K_i_*_(app)_. To confirm the specificity of fluorogenic substrate cleavage, samples of the digests were analysed by reverse-phased (RP)-HPLC on a Vydac 4.6 mm × 25 cm C_18_ RP-HPLC column, using fluorescence to detect the products of cleavage, as previously described (Blackman et al., 2002). Where appropriate, pronase (10 μg/ml) was added to digestion reactions to rapidly obtain complete substrate hydrolysis. As with many fluorogenic peptides, internal quenching and intermolecular dimerization effects were observed with all 6-IATR-labelled peptides at concentrations >5 μM, so it was not possible experimentally to determine accurate *K_m_* values for these substrates. However, cleavage rates were proportional to substrate concentrations up to 4 μM substrate, indicating that *K_m_* values are ≫4 μM (data not shown). For determination of cleavage specificity or relative cleavage rates of unlabelled *N*-acetylated peptides, partially-digested peptides (∼2 mM starting concentration) were fractionated by RP-HPLC eluting at 1 ml/min with either a 0–45% or 4.5−18% (v/v) gradient of acetonitrile in 0.1% TFA over 25 min. Digestion products were identified by electrospray mass spectrometry as described previously (Withers-Martinez et al., 2002; Yeoh et al., 2007; Child et al., 2010).

For assessing processing of native SERA5 by rPfSUB1, extracts containing native SERA5 were produced by hypotonic lysis of mature *P. falciparum* 3D7 schizonts into 25 mM HEPES, pH 7.4, 12 mM CaCl_2_, containing protease inhibitors, as described previously (Silmon de Monerri et al., 2011). Samples of extract were supplemented with either rPfSUB1 (1–10 U/ml), or purified rPfSUB1 PD (84 nM final concentration, to prevent cleavage by endogenous PfSUB1) (Jean et al., 2003), or both. Digestion reactions were allowed to proceed at 37 °C for up to 2 h. Samples taken at intervals were examined by Western blot, probing with the SERA5-specific monoclonal antibody 24C6.1F1 (Delplace et al., 1985), a kind gift of Jean-Francois Dubremetz, University of Montpellier 2, France.

### 2.4 Homology modelling of PfSUB1, PvSUB1, PkSUB1 and PbSUB1

PfSUB1 numbering used in this study refers to the *P. falciparum* 3D7 sequence (PlasmoDB ID PFE0370c; Supplementary Fig. S1) which differs from the *P. falciparum* T9/96 sequence (GenBank AJ002233; Blackman et al., 1998) by a deletion of two Ser residues in a polyserine stretch in the PD region. This makes the 3D7 sequence 688 residues in length, two residues shorter than the T9/96 sequence. Generation of our initial PfSUB1 homology model was described by Withers-Martinez et al. (2002). To check its validity and to search for improved models, the Modeller v9.3 suite (Eswar et al., 2008) was used. To obtain template structures, the 3D7 PfSUB1 sequence was submitted to pGenTHREADER within the PSIPRED server (http://bioinf.cs.ucl.ac.uk/psipred/). Homology models were generated on the basis of the top seven hits (PDB codes 1BH6, 1DBI, 1GCI, 1THM, 1R0R, 1MEE and 1TO2), using the Modeller package with varying numbers of template structures. The best models were selected on the basis of their DOPE score as calculated by the Modeller package, and on their stereochemical quality as calculated with Procheck (Laskowski et al., 1998). Homology models for PvSUB1, PkSUB1 and PbSUB1 were then generated based on the original PfSUB1 model, again using Modeller and Procheck (percentage amino acid identity with the PfSUB1 catalytic domain are: PvSUB1: 75%; PkSUB1: 76%; and PbSUB1: 68%; Supplementary Fig. S1). The root mean square deviation (RMSD) of Cα atoms between the four resulting models was <0.3 Å. Electrostatic calculations and all structural figures were generated with PyMOL (the PyMOL Molecular Graphics System, Version 1.3, Schrödinger LLC, Portland, USA, http://www.pymol.org/).

### 2.5 Modelling PfSUB1–peptide complexes

PfSUB1–peptide complexes were modelled on the basis of the crystal structures of subtilisin BPN′ and Carlsberg in complex with *Streptomyces* subtilisin inhibitor (PDB ID 2SIC; Takeuchi et al., 1991), eglin (PDB ID 1CSE; Bode et al., 1987; and 1SIB; Heinz et al., 1991), and chymotrypsin inhibitor 2 (PDB: 2SNI; McPhalen and James, 1988). The inhibitor-bound subtilisin structures and the PfSUB1 model were superimposed using Coot (http://www.biop.ox.ac.uk/coot/; Emsley et al., 2010), and each inhibitor truncated to a 10 residue segment (P5–P5′) spanning the enzyme active site pockets (Supplementary Fig. S2). The template residues were then replaced with the established PfSUB1 substrate sequence KITAQDDEES (SERA4st1) (Yeoh et al., 2007). The decapeptide was then manually adjusted in the active site of the PfSUB1 model using Coot to remove steric clashes. Polar hydrogens were added and the structure minimised by 500 steps of steepest descent minimisation using the modelling package Quanta 2006 (Accelrys Inc., San Diego CA, 2006) together with the CHARMM22 force field (MacKerell, 1998).

### 2.6 MD simulations

A MD simulation of PfSUB1 bound to the peptide substrate KITAQDDEES was performed with the Amber 11 suite of programs (Case et al., 2005) together with the ff99SB modifications (Simmerling et al., 2002; Hornak et al., 2006) of the Cornell et al. (1995) force field. The starting structure for the MD simulation was derived using the PfSUB1 homology model and modelling the peptide by superposition of the backbone coordinates of homologue complexes (PDB codes: 1LW6; Dauter et al., 1991; Radisky and Koshland, 2002; and 1MEE; Dauter et al., 1991). Missing side chains were built up by mutation of the corresponding amino acid using tleap from the Amber 11 package (Case et al., 2005). To avoid terminal charges on the peptides, the N- and C-terminal residues were capped with acetyl (ACE) and *N*-methylamine (NME) groups, respectively. The system was neutralised by adding sodium counter-ions and solvated in a truncated octahedron box of TIP3P (Jorgensen et al., 1983) water molecules, forming a solvent shell of at least 11 Å between each face of the box and the solute. The system was minimised by 250 steps of steepest descent minimisation followed by 250 steps of conjugate gradient minimisation. The particle mesh Ewald (PME) method (Cheatham et al., 1995) was used to treat long-range electrostatic interactions, and bond lengths involving hydrogen atoms constrained using the SHAKE algorithm (Ryckaert et al., 1977). The integration time step for the MD simulation was 2 fs, with a direct-space non-bonded cutoff of 9 Å. After minimisation, MD in the canonical ensemble (NVT) was carried out for 50 ps, during which the system was heated from 100 to 300 K. Harmonic restraints with force constants of 5 kcal/(mol Å^2^) were applied to all solute atoms in this step. Subsequent isothermal isobaric ensemble (NPT)-MD was performed for 50 ps to adjust the solvent density. Finally, the force constants of the harmonic restraints on the receptor atoms were gradually reduced to zero during 250 ps in the NVT ensemble. An additional 50 ps of unconstrained NVT-MD at 300 K with a time constant of 2.0 ps for heat bath coupling were performed to relax the system without constraints. The production run of the simulation achieved a length of 50 ns of which snapshots saved at 20 ps intervals were used for analysis. The ‘ptraj’ module of Amber 11 was used for analysing the root-mean square fluctuations (RMSF) about the mean position of atoms and formation of hydrogen bonds. For RMSF calculation, overall translational and rotational motions were removed with respect to the Cα-atoms of the PfSUB1 structure. Hydrogen bonds were defined by a distance cutoff of 3.2 Å and an angle cutoff of 120°.

### 2.7 Synthesis and purification of the peptidic alpha-ketoamides KS-182 and KS-466, and the peptidic aminoalcohol KS-378

Complete synthetic schemes for synthesis, purification and structural validation of these compounds are provided in Supplementary Data S1.

## 3 Results

### 3.1 Protease treatment of rPfSUB1 identifies an enzymatically active core domain with unaltered substrate specificity

**Fig. 1:**
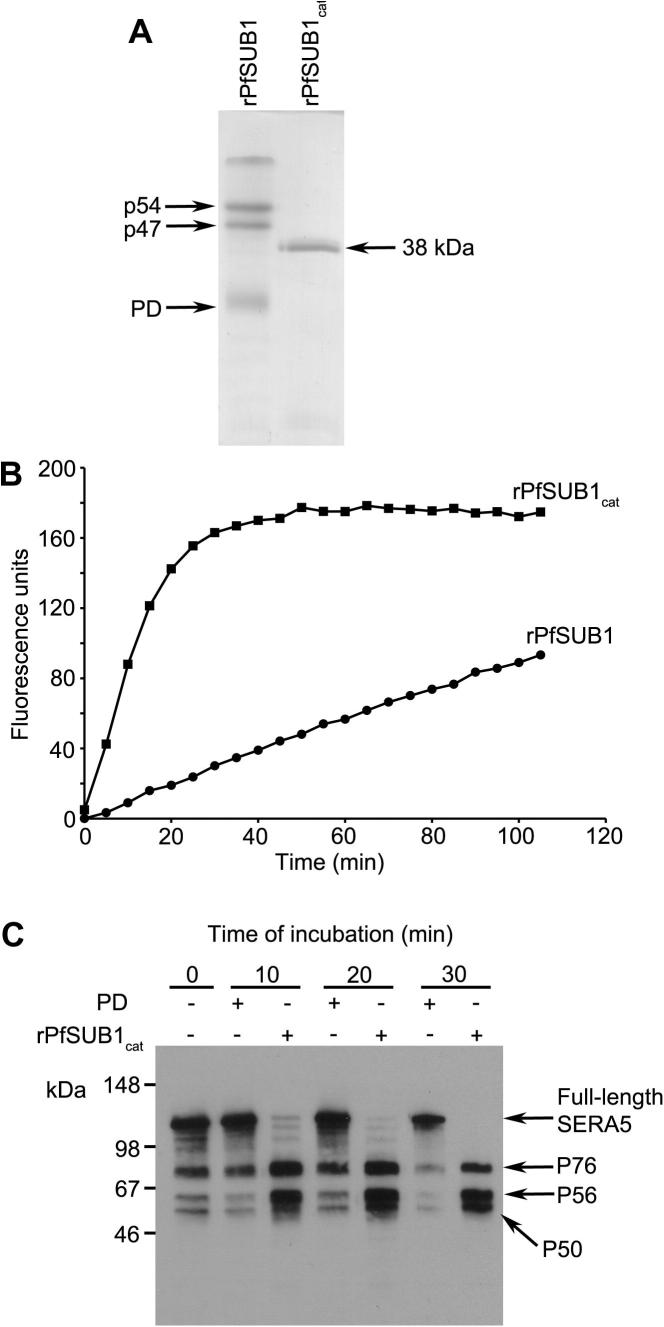
Production of an enzymatically active, soluble core domain of recombinant *Plasmodium falciparum* subtilisin-like protease 1 (rPfSUB1). (A) Limited chymotrypsin-treatment at 25 °C of insect cell-derived rPfSUB1 converts it to a relatively stable 38 kDa core domain (rPfSUB1_cat_) comprising Ser328 to Tyr671 (see Section 3.1). Note the disappearance of the prodomain in the treated sample, which was confirmed by Western blot with antibodies specific for the prodomain (not shown). (B) Progress curves showing cleavage of fluorogenic substrate SERA4st1F-6R12 by equivalent masses of rPfSUB1 and rPfSUB1_cat_. Substrate concentration in this assay was 0.1 μM, well below the *K_m_* for this substrate, which is ≫4 μM. The initial rate of cleavage mediated by rPfSUB1_cat_ was 9.8-fold faster than that mediated by untreated rPfSUB1. (C) Time-course showing processing in vitro of *P. falciparum*-derived serine rich antigen 5 (SERA5) by rPfSUB1_cat_. The full-length ∼120 kDa SERA5 is rapidly and correctly converted to the expected P76 and P56 forms in the presence of added rPfSUB1_cat_. Small amounts of P76 and P56, derived from endogenous PfSUB1 activity, are present in the starting (0 min) sample, but these levels do not significantly alter with time in samples supplemented with recombinant prodomain. Some conversion to the terminal SERA5 processing product P50 is also evident in the rPfSUB1_cat_-containing samples, presumably due to the presence in the parasite extracts of the cysteine protease responsible for this (Yeoh et al., 2007). No processing of SERA5 was observed in the presence of both rPfSUB1_cat_ and prodomain (not shown).

PfSUB1 is synthesised as a ∼82 kDa precursor zymogen which undergoes maturation in two sequential processing steps, both involving truncation from the N-terminus of the protein. In the first step, autocatalytic cleavage occurs at an internal _213_LVSAD ↓ NIDIS_222_ motif to produce a p54 species plus the PD, which remains in a tight complex with p54 and is a potent inhibitor of the cognate enzyme, as is common for subtilases (Sajid et al., 2000; Withers-Martinez et al., 2002). In the second processing step, further cleavage takes place at Asp249 to produce the p47 form, which accumulates in exonemes in the parasite. Both processing steps are recapitulated upon expression of rPfSUB1 in insect cells, but because the second step only takes place to a limited degree, the protein is secreted in the form of a heterogeneous mixture of the three products (Withers-Martinez et al., 2002). Both internal processing sites lie some distance N-terminal to the start of the PfSUB1 catalytic domain as defined by homology with bacterial subfamily S8A subtilisins, and therefore both p54 and p47 possess long N-terminal extensions of unknown function, as well as an unusual 26-residue C-terminal extension (Withers-Martinez et al., 2002). Previous attempts to obtain enzymatically active rPfSUB1 by expression of the predicted catalytic domain alone have proved unsuccessful, as expected (not shown) since the great majority of subtilisin-like enzymes absolutely require the PD for correct folding of the catalytic domain (Shinde and Thomas, 2011). We reasoned that, given the structural similarity of the PfSUB1 catalytic domain to the bacterial subfamily S8A subtilisins, it might be possible to use limited proteolytic digestion to convert insect cell-derived p54/p47 to a ‘core’ globular species comprising only the catalytic domain. This would provide a means to assess whether the catalytic domain of PfSUB1 possesses all of the structural determinants necessary for activity and substrate specificity. Purified rPfSUB1 was subjected to limited proteolytic digestion with trypsin, pancreatic elastase (data not shown) or chymotrypsin, then re-purified. As shown in Fig. 1A, chymotrypsin treatment efficiently and reproducibly converted the rPfSUB1 to a soluble, homogeneous ∼38 kDa product (named rPfSUB1_cat_), with a concomitant decrease in levels of the associated inhibitory PD (which was largely degraded by the treatment). Consistent with this, rPfSUB1_cat_ exhibited substantially increased proteolytic activity (Fig. 1B) whilst retaining its substrate specificity, as shown by examination of cleavage of both fluorogenic peptide substrates and unlabelled peptides (not shown), plus parasite-derived SERA5, an authentic protein substrate (Fig. 1C). N-terminal sequencing of rPfSUB1_cat_ gave homogeneous signals corresponding to the sequence Ser-Arg-Pro-Gly-Lys in the first five cycles of Edman degradation, unambiguously assigning its N-terminus as Ser328 (Supplementary Fig. S1). Electrospray mass spectrometric analysis of rPfSUB1_cat_ in the absence or presence of 100 mM DTT produced signals at *m*/*z* 38,328.3 and 38,333.5, respectively. This corresponds closely to PfSUB1 sequence extending from Ser328 to Tyr671 (calculated *m*/*z* 38,328.2 if six of the seven Cys residues are engaged in disulphide bonds, or *m*/*z* 38,334.2 in fully reduced form). This sequence is only slightly longer than that encompassed by our original homology model based on bacterial subtilisin catalytic domains (Phe339-Asn662; Supplementary Fig. S1) (Withers-Martinez et al., 2002). These results strongly suggest that all the structural features responsible for PfSUB1 activity and substrate specificity reside within the core catalytic domain of the protein.

### 3.2 Structural analyses of homology models reveal key interactions which are conserved in the active site cleft of four Plasmodium SUB1 orthologues

The first homology model of PfSUB1 was built in our laboratory almost 10 years ago based on the X-ray crystal structures of the four most closely homologous bacterial subtilisins available at that time (Withers-Martinez et al., 2002). Several new subtilisin crystal structures have subsequently become available, so we generated new homology models of PfSUB1 based on an extended number of templates (see Section 2.4 for further details). Encouragingly, all of the new models showed only marginal differences from the original PfSUB1 model in the core region, the overall RMSD of the backbone coordinates between the models deviating only by up to 0.6 Å, although there was much less deviation within and around the active site regions (data not shown). Only the loop regions, which are expected to be relatively flexible, show pronounced conformational changes and differ in their modelled length and composition. We therefore used the original PfSUB1 model for all subsequent analyses.

**Fig. 2:**
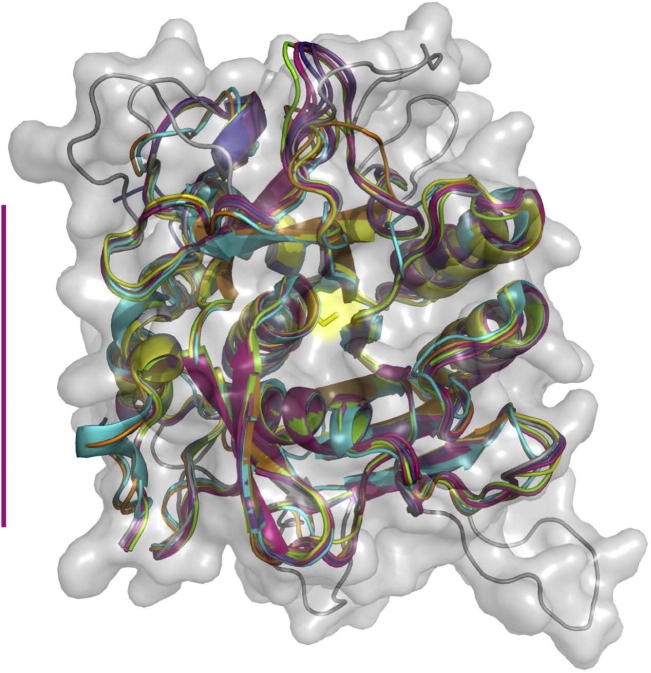
Structural model of the *Plasmodium falciparum* subtilisin-like protease 1 (PfSUB1) catalytic domain. Cartoon representation of the PfSUB1 homology model, which extends from *P. falciparum* 3D7 PfSUB1 residues 339–662, superimposed onto X-ray crystal structures of seven bacterial subtilisins. These are: subtilisin DY from *Bacillus licheniformis* (subtilisin Carlsberg) inhibited by *N*-benzyloxycarbonyl-Ala-Pro-Phe-chloromethyl ketone (PDB 1BH6, pink); *Bacillus* sp*. Ak.1* subtilisin (1DBI, light blue); *Bacillus lentus* subtilisin (1GCI, yellow); *Thermoactinomyces vulgaris* subtilisin (thermitase) (1THM, orange); *Bacillus licheniformis* subtilisin inhibited by turkey ovomucoid third domain (OMTKY3) (1R0R, green); *Bacillus mesentericus* subtilisin inhibited by eglin-C (1MEE, red); and subtilisin BPN′ from *Bacillus amyloliquefaciens* (subtilisin Novo) inhibited by chymotrypsin inhibitor 2) (1TO2, purple). The PfSUB1 homology model is depicted in its see-through molecular envelope (grey). The active site catalytic Ser side chain is shown as a yellow stick. The six surface ‘loops’ of PfSUB1, which are absent from the bacterial homologues, are clearly visible. The vertical purple bar on the left indicates a length of 30 Å.

Fig. 2 shows the PfSUB1 homology model superimposed onto the catalytic domain structures of the seven bacterial subtilisins used as templates for our homology modelling. The high degree of structural conservation within the core of the protein surrounding the catalytic Ser is immediately apparent. Variations are seen only on peripheral loops or strands, where six large insertions are clearly noticeable in PfSUB1 outside of a 15 Å radius sphere centred on the catalytic Ser. Despite their absence from homologous bacterial subtilisins, we have shown previously that individual deletion of five of these six PfSUB1 loops severely affects rPfSUB1 maturation and/or stability of the recombinant protein in our insect cell expression system, probably due to an impact on folding (Jean et al., 2005). Of importance for the present study, however, that analysis provided no experimental evidence that any of the loops contribute to PfSUB1 substrate specificity, and indeed examination of our model is in agreement with that, suggesting that all of the loops are disposed too far from the active site to impact on substrate binding.

**Fig. 3:**
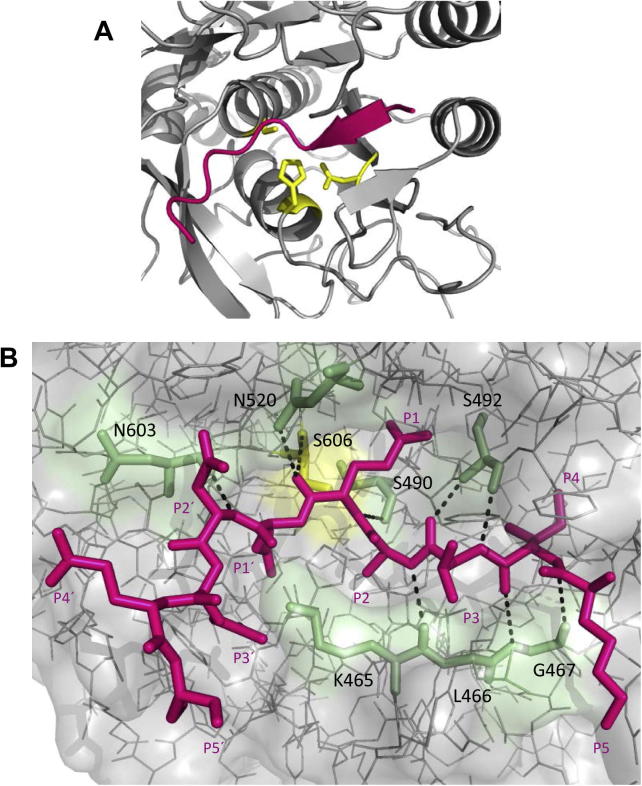
Structural model of *Plasmodium falciparum* subtilisin-like protease 1 (PfSUB1) in complex with its substrate reveals a canonical binding mode. (A) Cartoon representation of PfSUB1 (grey), zoomed on the active site, showing that canonical binding of the SERA4st1 decapeptide substrate KITAQDDEES (P5–P5′, arranged right to left across the image, pink) in the catalytic groove results in the formation of a two-stranded antiparallel β-sheet. Residues forming the catalytic triad (Ser606, His428 and Asp372) are shown as yellow sticks. (B) Atomic level illustration of the backbone interactions made by SERA4st1 residues at positions P1 (Gln), P2 (Ala), P3 (Thr), P4 (Ile) and P2′ (Asp) with residues in the active site pockets S1 (Ser606 and Ser490), S2 (Lys465), S3 (Ser492), S4 (Gly467) and S′ (Asn603), with these PfSUB1 residues represented as pale green sticks and the hydrogen bonding pattern shown as dotted lines. The PfSUB1 oxyanion hole partner Asn520, which helps to stabilize the oxyanion generated in the tetrahedral transition state, is also pictured. In addition to the canonical binding, side-chains of SERA4st1 residues at positions P1′ (Asp) and P3′ (Glu) could potentially contribute to the substrate–enzyme binding by interacting with the PfSUB1 S2 Lys465 side chain ε-amino group. In bacterial family S8 subtilisins, this Lys is replaced by a conserved Gly. The catalytic Ser is shown in yellow.

A previous side-by-side comparison of peptide substrates based on experimentally-confirmed and predicted PfSUB1 cleavage sites (Supplementary Fig. S3) identified the most efficiently-cleaved peptide to be an *N*-acetylated peptide corresponding to a cleavage site in SERA4, KITAQ ↓ DDEES (here called SERA4st1) (Koussis et al., 2009). Fig. 3 shows a structural model of the SERA4st1–PfSUB1 complex. The substrate (shown in non-*N*-acetylated form) lies in an extended conformation, the main-chain of the P4–P1 residues in its N-terminal half forming the upper strand of a two-stranded antiparallel β-sheet involving PfSUB1 residues Lys465-Gly467 below the groove (Fig. 3A and B). This mode of substrate binding is typical of subtilisins (Supplementary Fig. S2), and results in the P1–P4 segment of the substrate being held relatively tightly in the groove. Other canonical hydrogen-bonding interactions include between the P3 main chain and Ser492 (which also contributes to the polar nature of the PfSUB1 S1 pocket via its side-chain – see below), between the P2′ backbone and Asn603, and between the carbonyl oxygen of the P1 Gln and the oxyanion hole residue Asn520. Of additional interest is the observation that the side-chains of two prime side substrate residues, P1′ Asp and P3′ Glu, are in a position to interact with the side-chain ε-amino group of Lys465, which forms one end of the lower PfSUB1 β-strand described above and which also is a prominent component of the S2 pocket.

**Fig. 4:**
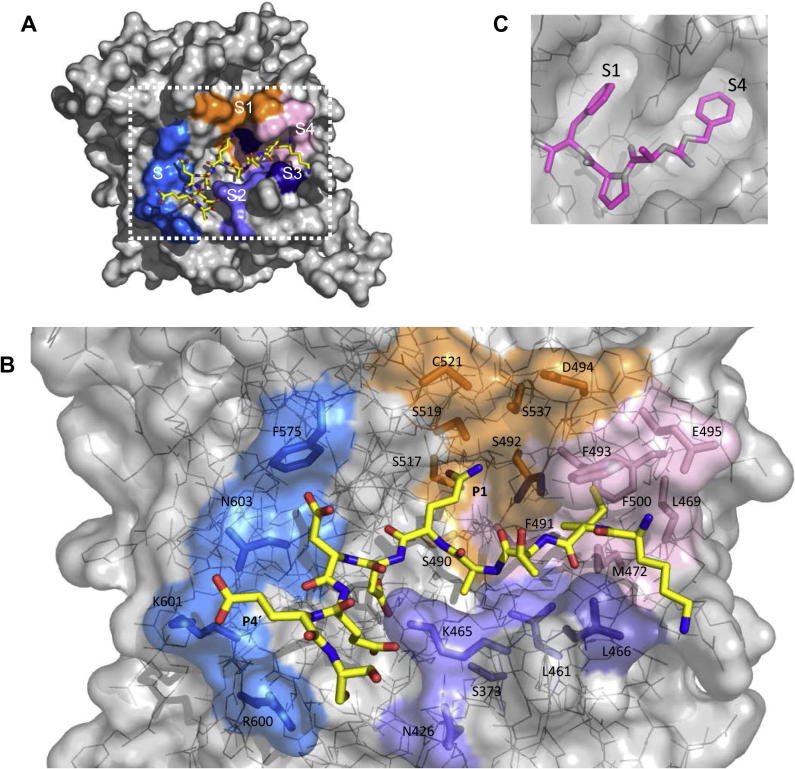
Architecture of the *Plasmodium falciparum* subtilisin-like protease 1 (PfSUB1) active site. (A) Molecular surface representation of PfSUB1 (grey), in complex with SERA4st1 (yellow carbon coloured stick). The five substrate binding pockets comprise S1 (orange), S2 (slate blue), S3 (indigo blue), S4 (pink) and S′ (turquoise). (B) Close-up view of the active site (delimited in (A) by the white dotted frame) using a see-through PfSUB1 molecular surface to reveal residues that form the substrate-binding pockets, colour-coded as in (A). These are: in the S1 pocket: Ser517, Ser519, Ser490, Ser492, Asp494, Ser537 and Cys521; in the S2 pocket: Ser373, Asn426, Leu 461 and Lys465; in the S3 pocket: Leu466 and Ser492; in the S4 pocket: Leu469, Met472, Phe491, Phe493, Glu 495 and Phe500; and in the S′ pocket: Phe575, Arg600, Lys601 and Asn603. Ser492 is seen to belong to two pockets; its side chain is part of S1 and its backbone S3. This position corresponds to a Gly in several bacterial subtilisins. As seen in this view, the S1 pocket is polar, the S2 pocket is small due to the Lys465 side chain conformation, the S3 pocket faces the solvent, the S4 pocket is non-polar (with the exception of Glu495 at the rim of the pocket), and the S′ pocket is mainly basic. (C) For comparison, molecular surface of the bacterial subtilisin DY Carlsberg (1BH6) in grey, shown with bound *N*-benzyloxycarbonyl-Ala-Pro-Phe-chloromethyl ketone (pink sticks), showing the S1 and S4 pockets that dictate specificity. Both pockets are solvent-accessible, large and predominantly hydrophobic, explaining the broad specificity of this enzyme and its preference for large non-polar residues at the P1 and P4 positions.

For most S8A subfamily subtilisins, enzyme specificity is primarily dictated by interactions of the P1–P4 residue side-chains with the corresponding S1–S4 active site pockets (Siezen and Leunissen, 1997). Fig. 4 depicts the distribution of the various PfSUB1 active site pockets and their constituent residues, in association with the SERA4st1 substrate. The enzyme S4 pocket (indicated in pink in Fig. 4A and B) is well defined and partially buried as it extends into the core of the protein. It is characterised by a lining of hydrophobic residues, involving PfSUB1 residues Leu469 which lies at the pocket entrance, plus Met472, Phe491, Phe493 and Phe500, and is well suited to the aliphatic residues preferred at the P4 position (Supplementary Fig. S3). The outer edge of the S4 pocket is formed by Glu495, which may help to stabilize basic residues in the P5 position (see below). In common with the S8A subfamily bacterial subtilisins, the PfSUB1 S3 pocket is not well defined, and the side chain of the P3 residue is directed out towards the solvent with the only significant interactions being the above-mentioned main-chain interactions with Ser492 (Fig. 3). This likely explains the relative lack of restriction at the substrate P3 position (Supplementary Fig. S3). The most obvious characteristic of the S2 pocket is that it is small, being delimited by the side-chain of Lys465 (which is a Gly in most subfamily S8A subtilisins; see Supplementary Table S1), explaining the strict restriction to only the small residues Ala or Gly at the P2 position (Supplementary Fig. S3). The PfSUB1 S1 pocket (orange) is narrower than the equivalent subtilisin Carlsberg pocket (Fig. 4C) and is characterised by a cluster of five polar residues, Ser490, Ser492, Ser517, Ser519 and Ser537. The latter residue corresponds to Gly166 in bacterial subfamily S8A subtilisins, which lies at the bottom of the S1 pocket and has been extensively mutated to demonstrate its importance in enzyme substrate preference and catalysis; for example replacement with Asp of Gly166 in the subtilisin BPN′ S1 pocket modifies its specificity towards substrates containing P1 Lys (Wells et al., 1987b). The rim of the PfSUB1 S1 pocket is formed by Asp494 plus a free cysteine, Cys521, which also increases the polarity of the S1 pocket and may account for the sensitivity of PfSUB1 to para-hydroxymercuribenzoate (pHMB) and other sulfhydryl-reactive compounds (Withers-Martinez et al., 2002). In most bacterial subtilisins, the residue at this position (the structurally equivalent residue in subtilisin Carlsberg is Ser156; Supplementary Table S1) also dramatically influences P1 preference (e.g. Wells et al., 1987a,b). On the PfSUB1 S′ sub-site, the top rim of the S′ pocket is formed by Phe575, which is highly conserved in subfamily S8A bacterial subtilisins. Two of the three other residues (Arg600, Lys601 and Asn603) making up the PfSUB1 S′ pocket are basic. Together with the influence of Lys465, this results in an overall highly basic character to the S′ surface.

### 3.3 Molecular dynamics simulation suggests that both prime and non-prime side interactions are involved in substrate recognition by PfSUB1

**Fig. 5:**
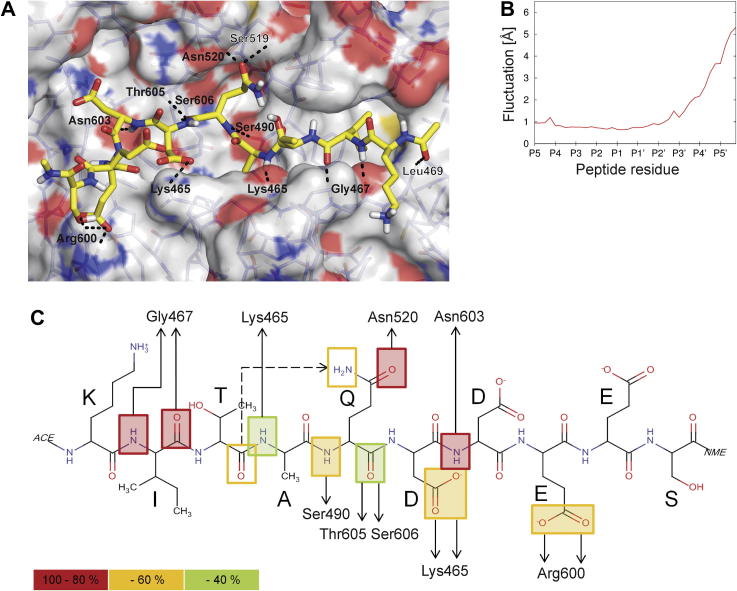
Molecular Dynamics simulation of the *Plasmodium falciparum* subtilisin-like protease 1 (PfSUB1) structure in complex with SERA4st1. (A) Binding mode of SERA4st1 extracted after 40 ns from the MD simulation. Hydrogen bond interactions between the peptide (yellow carbon coloured stick) and PfSUB1 are indicated by dashed lines. (B) Root-mean square fluctuations (RMSF) about the mean position of the peptide backbone atoms. For RMSF calculation, overall translational and rotational motions were removed with respect to the Cα atoms of the PfSUB1 structure. (C) Scheme of hydrogen bonds formed during the MD simulation between PfSUB1 and peptide backbone and side-chain atoms. The peptide orientation is shown from N- to C-terminus left-to-right, and thus is opposite to the orientation shown in (A). Very strong hydrogen bonds (occupancy of 80–100%) are boxed in red, strong hydrogen bonds (occupancy of 60–80%) are in orange, and weaker hydrogen bonds (occupancy of 40–60%) are shown in green. Occupancy values were obtained over the course of the entire 50 ns of simulation time. Repeating the analysis for the first and second half of the trajectory resulted in the same hydrogen bond pattern.

To seek further insights into the binding determinants of the SERA4st1 substrate, we performed an unrestrained MD simulation of the PfSUB1–SERA4st1 complex in explicit solvent. Determining the RMSF of backbone atoms of the bound peptide revealed that pronounced conformational changes occur at the P2′–P5′ prime side residues, whereas the remaining part of the peptide backbone remain essentially unchanged (Fig. 5A and B). The restricted fluctuation of the P5–P1′ residues is in agreement with strong backbone-backbone hydrogen bonds being formed between P2′–P4 and the PfSUB1 binding site (Fig. 3). Over the course of the 50 ns of simulation time, very stable canonical hydrogen bonds (with an occupancy of at least 80%, indicated in red in Fig. 5C) are formed between the amino group of P2′ and the carbonyl group of Asn603 in the S2′ pocket as well as between the amino and carbonyl group of P4 with the corresponding backbone groups of Gly467. Further canonical hydrogen bonds (with an occupancy of at least 40%, indicated in orange or green in Fig. 5C) are formed between the carbonyl group of P1 and Thr605 and Ser606, respectively, between the amino group of P1 and Ser490, and between the amino group of P2 and Lys465, respectively. In addition, stabilizing hydrogen bond interactions are formed between the side-chain carboxylate group of P1 and the backbone amide group of Asn520 in the S1 pocket, between the side-chain keto group of P1′ and the side chain ε-amino group of Lys465 in the S2 pocket, and between the side-chain hydroxyl group of P3′ and the side-chain guanidinium group of Arg600.

All of these observations confirm the canonical binding mode suggested by our initial modelled peptide–PfSUB1 complex structure. In addition, they suggest that stabilizing prime side interactions, in particular involving the P1′ and P3′ residues, may also play important roles in substrate binding and correct positioning of the cleavage site. This is of particular interest as it could explain the observed preference of PfSUB1 for substrates possessing acidic or hydroxyl-containing residues on their P′ side.

### 3.4 Cleavage of PfSUB1 substrates requires interactions with prime side residues

Our earlier alanine scanning studies (Koussis et al., 2009) using a panel of derivatives of a quenched fluorogenic substrate based on the internal PfSUB1 autocatalytic cleavage site _213_LVSAD ↓ NIDIS_222_, showed that replacement with Ala of the P3′ Asp had a significant effect on cleavage efficiency, reducing the *k*_cat_/*K_m_* by ∼50%. Taken together with the high frequency of acidic and/or hydroxyl-containing side-chains in the prime side positions of physiological PfSUB1 substrates (Supplementary Fig. S3), and the MD data which suggest stabilising interactions between PfSUB1 residues and the P1′ and P3′ residues, we wished to further address the possibility that prime side interactions might play an unusually important role in substrate recognition by PfSUB1 and its orthologues by examining the activity of PfSUB1 against peptide substrates depleted of or entirely lacking prime side residues. Peptide Ac-EIKAET, based on the SERA5 site 1 peptide Ac-EIKAE ↓ TEDDD previously shown to be an excellent PfSUB1 substrate (Yeoh et al., 2007), was examined for its capacity to be cleaved by rPfSUB1_cat_. No hydrolysis of Ac-EIKAET was evident even following prolonged incubations (data not shown), indicating that a single prime side residue is insufficient for efficient interaction with PfSUB1. Many commonly-used fluorogenic protease substrates are based on peptidyl derivatives of 7-amino-4-methylcoumarin (AMC), in which cleavage of the peptide–AMC anilide bond results in release of the free fluorophore. These substrates are widely used for activity assays with subtilisins and other proteases (e.g. Backes et al., 2000; Wildeboer et al., 2009), but their utility depends on the enzymes under examination not having essential prime side requirements. Peptide Ac-KITAQ-AMC, based on the non-prime side P5–P1 residues of the SERA4st1 substrate used for the modelling experiments, was next examined for its capacity to act as a PfSUB1 substrate. Again, this was completely resistant to hydrolysis (not shown). Although alternative possibilities cannot be ruled out – such as steric hindrance imposed by the bulky AMC moiety perturbing interactions with both sides of the scissile bond – these findings are consistent with the MD data in suggesting that prime side interactions are important for substrate recognition by PfSUB1.

**Fig. 6:**
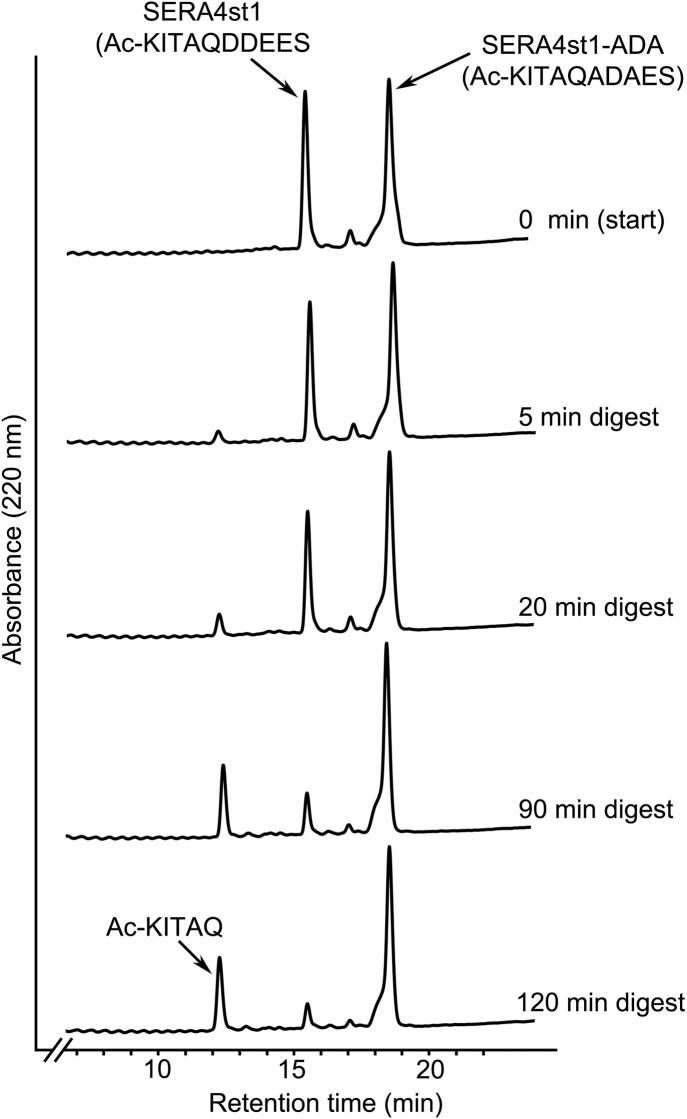
Prime side residues play a critical role in substrate recognition by *Plasmodium falciparum* subtilisin-like protease 1 (PfSUB1). Reversed-phase-HPLC chromatograms showing a time-course of digestion by rPfSUB1_cat_ of an equimolar mixture of the *N*-acetylated decapeptide SERA4st1 (Ac-KITAQ ↓ DDEES; based on a PfSUB1 processing site in SERA4) plus peptide SERA4st1-ADA, in which the P1′ and P3′ residues of SERA4st are replaced with Ala. The top chromatogram shows the elution profile of the undigested mixture, whilst the lower chromatograms are of samples taken over the course of a 120 min digestion. Identities of substrate peaks are indicated, as is the N-terminal product of SERA4st1 cleavage (Ac-KITAQ) (determined by electrospray mass spectrometry). As described previously (Yeoh et al., 2007) the highly polar C-terminal cleavage product of SERA4st1 digestion is not retained by the Reversed-phase-HPLC column but elutes in the column flow-though. Note that for determination of relative initial rates of cleavage, cleavage rates were compared when the faster-cleaved peptide (SERA4st1) was digested by ⩽20%. For clarity, in this experiment digestion was allowed to proceed much further. As can be seen, by 120 min, the SERA4st1 peak intensity was reduced by 84.6%, whilst the SERA4st1-ADA peak was reduced by only ∼1%.

In a third approach to investigating the role of prime side interactions in PfSUB1–substrate recognition, we produced a modified form of the SERA4st1 peptide substrate (called SERA4st1-ADA) in which both the P1′ and P3′ residues were replaced by Ala. Based on our MD data, these substitutions would be predicted to abolish potentially important stabilizing interactions with the S′ surface of the enzyme. To compare cleavage efficiencies of SERA4st1 and SERA4st1-ADA, we used a previously-described method in which an equimolar mixture of the two peptides was supplemented with rPfSUB1_cat_ and the rates of peptide hydrolysis established by RP-HPLC analysis of the resulting digestion products (Child et al., 2010). Under such conditions, because the peptides effectively act as competing substrates, their relative initial rates of hydrolysis are solely a function of the ability of PfSUB1 to discriminate between them, and so are directly proportional to their relative *k*_cat_/*K_m_* values, irrespective of their concentration (Cornish-Bowden, 2004). As shown in Fig. 6, this assay demonstrated that SERA4st1-ADA is an extremely poor substrate for PfSUB1, exhibiting an initial cleavage rate at least 80-fold lower than that of the parental SERA4st1 peptide (based on comparison of areas under substrate peaks when digestion of SERA4st1 had proceeded ⩽20%). Importantly, although prolonged digestion of SERA4st1-ADA alone resulted in only limited cleavage, this took place predominantly at the expected Gln-Ala bond, as shown by the appearance in the RP-HPLC elution profile of the same N-terminal cleavage product (Ac-KITAQ) as seen with SERA4st1 (Supplementary Fig. S4). These results show unambiguously that prime side residues play a critical role in substrate hydrolysis by PfSUB1.

### 3.5 Homology modelling and recombinant expression of other Plasmodium SUB1 orthologues defines conserved and species-specific characteristics

**Fig. 7:**
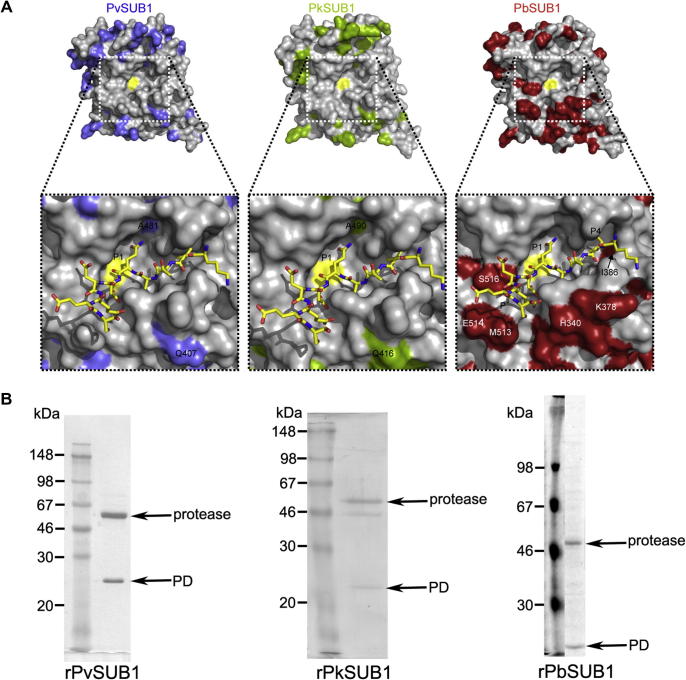
Homology modelling and recombinant expression reveals similarities and differences between *Plasmodium* subtilisin-like protease 1 (SUB1) orthologues. (A) Molecular surface representation of homology models of the *Plasmodium vivax* (Pv)SUB1, *Plasmodium knowlesi* (Pk)SUB1 and *Plasmodium berghei* (Pb)SUB1 catalytic domains. Coloured residues (blue for PvSUB1, green for PkSUB1, red for PbSUB1) indicate positions that are different from structurally equivalent residues in PfSUB1, whereas identical residues are shown in grey. The catalytic Ser is shown in yellow. White dashed frames delimitate the active site grooves, and the zoomed views below show the modelled binding mode of SERA4st1 (KITAQ ↓ DDEES). PvSUB1 and PkSUB1 are almost identical to PfSUB1 in the active site region, with the only two substitutions being Ala481 and Gln407 in PvSUB1, and Ala490 and Gln416 in PkSUB1. In contrast, PbSUB1 diverges significantly, with six substitutions (His340, Lys378, Ile386, Met513, Glu514 and Ser516), indicating likely differences in the specificity of this enzyme at the P4 and P′ positions. (B) Recombinant expression of SUB1 orthologues. Proteins were expressed using baculovirus-infected insect cells and purified as described in Section 2.1. Positions of migration of the protease domain and the prodomain species resulting from cleavage during maturation are indicated. The left-hand track on each gel contains molecular mass standards of the indicated sizes. Predicted molecular masses of the protease domains resulting from cleavage at the expected autocatalytic processing site (Supplementary Fig. S1) are: PvSUB1, 48,082 Da; PkSUB1, 49,072 Da; and PbSUB1, 44,868 Da.

To investigate potentially informative structural similarities and differences between the active site architectures of SUB1 orthologues from other clinically and experimentally important malarial species, we built homology models of the catalytic domains of PkSUB1, PvSUB1 and PbSUB1, for comparison with the PfSUB1 model. This revealed (Fig. 7A) that the active sites of PvSUB1 and PkSUB1 are identical to each other and very similar to that of PfSUB1, the only important difference being the replacement of Ser537 at the base of the PfSUB1 S1 pocket with Ala in PvSUB1 and PkSUB1 (see also Supplementary Table S1). This would be predicted to result in a slightly less polar S1 pocket in these enzymes. In addition, Glu463 below the PfSUB1 S2 pocket is replaced by a Gln residue in PvSUB1 and PkSUB1, but this lies some distance from the substrate interactions so it is anticipated to have little if any effect on the conformation of the S2 pocket. In contrast, an examination of the modelled PbSUB1 active site indicated more structural divergence, with a total of six substitutions relative to PfSUB1. Notable amongst these is Ile386 at the base of the S4 pocket, which replaces a Met residue in PfSUB1, PvSUB1 and PkSUB1, and results in a slight increase in the size and hydrophobicity of the PbSUB1 S4 pocket. The PbSUB1 S′ sub-site composition is even more divergent, with Ser516 replacing Asn603 in PfSUB1, and Met513 and Glu514 replacing, respectively, Arg600 and Lys601 in PfSUB1. Combined, these substitutions are predicted to provide PbSUB1 with a less basic S′ surface than the other three orthologues, evident from a comparison of the electrostatic surface views (Supplementary Fig. S5). In summary, modelling of the SUB1 orthologues suggests that PvSUB1 and PkSUB1 likely share very similar substrate specificity with PfSUB1, whilst some differences in the specificity of PbSUB1 at the P4 and P′ positions are likely.

To test this interpretation, we expressed recombinant PvSUB1, PkSUB1 and PbSUB1 in the same baculovirus expression system previously used for PfSUB1. As expected, examination of the purified expressed products suggested that, similar to PfSUB1, each was secreted in a complex with its cognate PD (Fig. 7B), as indicated by the presence of a 25–30 kDa species observable on Coomassie-stained gels of the purified proteins in addition to the higher-migrating product of around the expected mass for the catalytic domain-containing protein product. This was most evident in the case of rPvSUB1, where the putative PD and catalytic domain species were sufficiently highly-expressed to allow analysis of the latter by N-terminal sequencing. This identified the N-terminal sequence Asp-Val-Ser-Leu-Ala in the first five cycles of Edman degradation, confirming its identity and consistent with autocatalytic cleavage at the predicted internal processing site (Supplementary Fig. S1). Preliminary experiments showed that the purified products exhibited low protease activity in their PD-bound form, so all were subjected to limited digestion with chymotrypsin as described above for PfSUB1; this successfully resulted in each case in conversion to a lower molecular mass form and complete or partial removal of the PD species as determined by Coomassie-blue staining (not shown). The resulting ‘activated’ protease preparations were then examined for their capacity to cleave the PfSUB1 substrate SERA4st1F6R-12 (a fluorogenic substrate based on P5–P5′ of the SERA4st1 cleavage site), as well as SERA5st1F-6R and SERA5st2F-6R, fluorogenic substrates based on P4–P4′ of the SERA5 site 1 and site 2 processing sites, IKAE ↓ TEDD and IFGQ ↓ DTAG (Yeoh et al., 2007). All three enzymes cleaved these substrates at the same Gln-Asp, Glu-Thr and Gln-Asp bonds, respectively, that are cleaved by PfSUB1, as determined from the characteristic elution profiles of the digestion products on RP-HPLC (not shown). In the absence of an active-site probe, we were unable to accurately determine concentrations of the active forms of the different recombinant enzymes, and thus could not calculate *k*_cat_ values for cleavage of the substrates. However, we noticed that the activity of rPbSUB1 against all three substrates was consistently at least 10-fold lower than expected from concentrations of this protein as determined by Coomassie blue-stained gels (not shown). In the light of this and the modelling data suggesting that PbSUB1 may possess divergent substrate specificity, we decided to explore this in more detail.

An examination of the predicted internal processing site within PbSUB1 (Supplementary Fig. S1) shows that, unlike PfSUB1, PvSUB1 or PkSUB1, the PbSUB1 cleavage site does not contain an acidic residue in either the P1′ or P3′ positions, instead possessing P1′ Ser. A P1′ Ser is found in some known PfSUB1 and predicted PvSUB1 and PkSUB1 substrates, but is strikingly conserved in most predicted PbSUB1 target sites, including all four known sites in the *P. berghei* MSP1 (PbMSP1) (Jennings et al., 1998) and many of the predicted cleavage sites in *P. berghei* SERA family members, including PbSERA3, thought to be essential in *P. berghei* blood-stages (Putrianti et al., 2010; Arisue et al., 2011) (Supplementary Table S2). To confirm that PbSUB1 can cleave substrates based on some of these sites, unlabelled *N*-acetylated decapeptides corresponding to three of the established cleavage sites within PbMSP1, as well as a predicted cleavage site within PbSERA3 (the *P. berghei* orthologue of *P. falciparum* SERA6) were examined for their capacity to be correctly cleaved by rPbSUB1. As shown in Supplementary Table S3, peptides Ac-TTSGQ ↓ SSTEP, Ac-VVTGE ↓ SEETS, Ac-TTRAE ↓ SEEDI and Ac-DVSGQ ↓ SENHQ were all correctly cleaved by rPbSUB1 at the expected positions, as shown by RP-HPLC examination of digestion products.

**Fig. 8:**
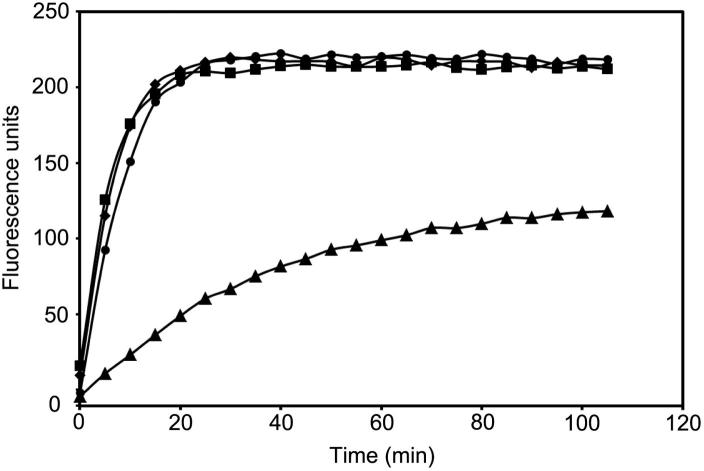
*Plasmodium berghei* subtilisin-like protease 1 (PbSUB1) prefers a P1′ Ser to Asp. Typical progress curves showing cleavage of fluorogenic substrate SERA4st1F-6R (6-iodoacetamido tetramethylrhodamine (6-IATR)-labelled Ac-CITAQDDEEC) by recombinant *Plasmodium falciparum* SUB1 catalytic domain (rPfSUB1_cat_; closed circles) and rPbSUB1_cat_ (closed triangles), and cleavage of SERA4st1F-6R-S (6-IATR-labelled Ac-CITAQSDEEC) by rPfSUB1_cat_ (closed squares) and rPbSUB1_cat_ (closed diamonds). Substitution of the P1′ Asp in SERA4st1F-6R with a Ser in SERA4st1F-6R-S results in a 5.8-fold increase in initial hydrolysis rates by rPbSUB1_cat_, but has no significant effect on cleavage by rPfSUB1_cat_. Both substrates were used at 0.1 μM final concentration in the digestion reactions.

To address the potential importance of the P1′ Ser residue in substrate cleavage by PbSUB1, we explored whether a PfSUB1 substrate that is a poor substrate for PbSUB1 could be converted to a better PbSUB1 substrate by substitution of the P1′ residue with Ser. We produced the fluorogenic substrate SERA4st1F-6R, which is the peptide Ac-CITAQDDEEC (corresponding to P4–P4′ of the SERA4st1 cleavage site) labelled on both terminal cysteine side-chains with 6-IATR. In parallel, we generated the derivative SERA4st1F-6R-S, which contains a Ser substitution of the P1′ (Asp) residue in SERA4st1F-6R. Cleavage of SERA4st1F-6R by rPfSUB1_cat_ resulted in the expected florescence increase, with conversion of the substrate to just two fluorescent products corresponding to cleavage at the expected Gln-Asp bond, as determined by RP-HPLC and mass-spectrometry (data not shown). Fig. 8 shows a kinetic analysis comparing cleavage of SERA4st1F-6R and SERA4st1F-6R-S by rPfSUB1_cat_ and rPbSUB1_cat_. At a standard concentration of both substrates (0.1 μM) which is well below their *K_m_* and therefore corresponding to conditions where the rate of cleavage is directly proportional to *k*_cat_/*K_m_*, it can be seen that whereas rPfSUB1_cat_ cleaves both substrates equally well, SERA4st1F-6R-S is a much better substrate for rPbSUB1_cat_ than SERA4st1F-6R. These results confirm that – as in the case of PfSUB1 – prime side resides do indeed contribute to substrate recognition by PbSUB1, and suggest that PbSUB1 prefers P1′ Ser to Asp, whereas PfSUB1 does not discriminate between them under these conditions. The observation that authentic PbSUB1 cleavage sites in at least two essential proteins possess P1′ Ser suggests that the enzyme has co-evolved with its cognate endogenous substrates in order to maintain optimal catalytic activity.

### 3.6 Peptidyl alpha-ketoamides based on a PfSUB1 substrate inhibit SUB1 from all three major human pathogens

**Fig. 9:**
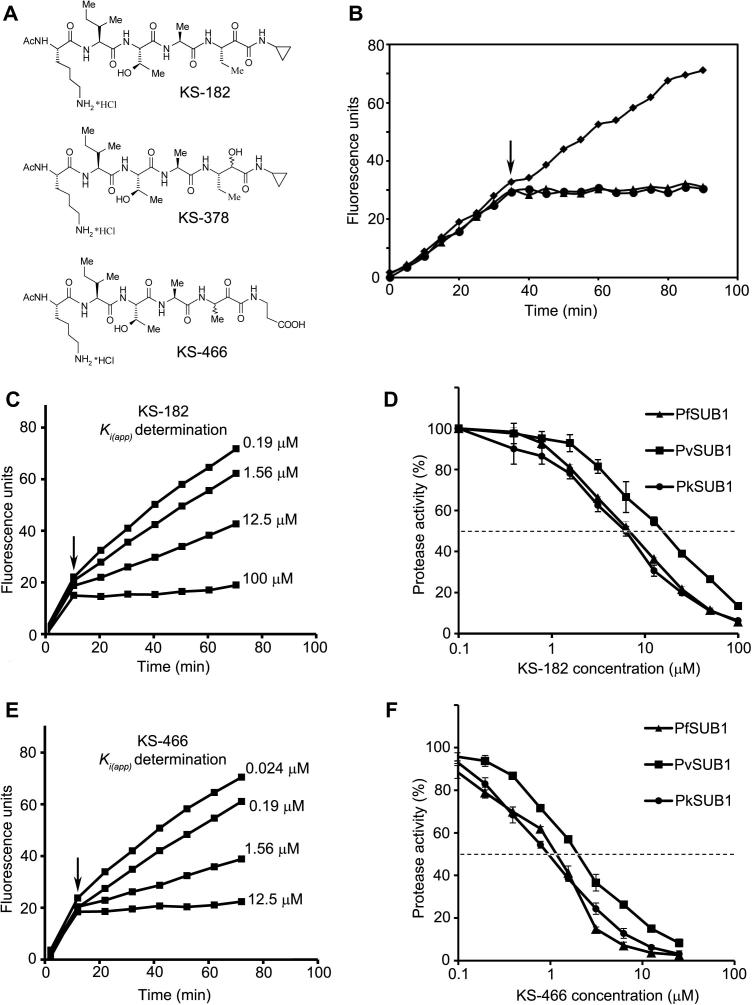
Peptidyl alpha-ketoamides based on a *Plasmodium falciparum* subtilisin-like protease 1 (PfSUB1) substrate are micromolar inhibitors of PfSUB1, *Plasmodium vivax* SUB1 (PvSUB1) and *Plasmodium knowlesi* SUB1 (PkSUB1). (A) Structures of the alpha-ketoamide KS-182, the corresponding aminoalcohol KS-378, and the extended KS-182 derivative KS-466. (B) Typical progress curve showing the profile of fluorescence increase with time produced by cleavage of substrate SERA4st1F-6R12 (0.1 μM) by recombinant *P. falciparum* SUB1 catalytic domain (rPfSUB1_cat_; 1 U/ml). The arrow indicates the point at which the reactions were supplemented with KS-182 (100 μM final, added from a 10 mM stock solution in DMSO; closed triangles), 1 mM para-hydroxymercuribenzoate (positive control inhibitor, closed circles), or 1% v/v DMSO (vehicle only control, closed squares). (C) and (E) Determination of *K_i_*_(app)_ values for inhibition of rPfSUB1_cat_ by KS-182 and KS-466. Progress curves showing the effects of adding different concentrations of the peptidyl alpha-ketoamides to an ongoing digestion are as in A. The point of compound addition is indicated by an arrow in each plot. In all cases, compounds were diluted 1:100 into the digestion reactions, from stock dilutions in DMSO. The final DMSO concentration in each reaction was therefore invariant at 1% (v/v). Values of *K_i_*_(app)_ were calculated as described in Section 2.3. Since the concentration of substrate SERA4st1F-6R12 used for these experiments (0.1 μM) is well below its *k_m_*, the calculated *K_i_*_(app)_ is unlikely to be significantly different from the *K_i_* (Nicklin and Barrett, 1984). (D and F) Dose–response curves showing the relationship between concentrations of KS-182 or KS-466, and inhibition of rPfSUB1_cat_, rPkSUB1_cat_, and rPvSUB1_cat_. Calculated IC50 values are 6 μM, 6 μM and 12 μM, respectively, for KS-182, or 1 μm, 1 μM and 2 μM for KS-466. Points are means of triplicate measurements and error bars indicate S.D. values. The horizontal dotted line in each plot indicates 50% inhibition of protease activity.

Our evidence that PfSUB1, PvSUB1 and PkSUB1 share similar substrate specificity suggested that it might be possible to design substrate-based compounds that inhibit all three proteases. Peptide alpha-ketoamides are well documented as mechanism-based covalent inhibitors of serine proteases, typically binding in a substrate-like manner and forming reversible covalent bonds with the hydroxyl side-chain of the catalytic Ser, so to test that notion we synthesised two *N*-acetylated peptidyl alpha-ketoamides based on the structure of the best known PfSUB1 substrate, SERA4st1. Compound KS-182 (Fig. 9A) was first examined for its capacity to inhibit rPfSUB1_cat_ activity. As shown in Fig. 9B, addition of KS-182 (final concentration 100 μM) to a digestion reaction resulted in rapid and essentially complete inhibition of substrate hydrolysis, indicating that KS-182 is a fast-binding inhibitor of PfSUB1. Further similar progress-curve experiments (Fig. 9C) allowed us to calculate an apparent tight binding inhibition constant (*K_i_*_(app)_) of 7.3 μM for inhibition of rPfSUB1_cat_. This was confirmed in additional dose–response experiments (Fig. 9D) which showed that the IC50 for steady-state inhibition of rPfSUB1_cat_ by KS-182 is ∼6 μM. Importantly, KS-182 also inhibited rPvSUB1 and rPkSUB1 with IC50 values of ∼12 μM and ∼6 μM, respectively. In accord with the distinct substrate requirements of PbSUB1, the IC50 of KS-182 against PbSUB1 was much higher, at ⩾100 μM (not shown). As a control compound for these experiments we produced a derivative of KS-182 called KS-378, in which the ketoamide function is replaced by an amino-alcohol group (Fig. 9A); this is expected to lack the capacity to form a covalent adduct with the enzyme catalytic Ser. KS-378 showed no inhibitory activity against any of the SUB1 orthologues at concentrations up to 100 μM (data not shown).

Encouraged by our results with KS-182, we designed an analogue possessing an extended terminal structure designed to mimic the prime side interactions predicted by our modelling and experimental data to be important for substrate recognition by PfSUB1. Compound KS-644 (Fig. 9A) possesses a carboxyl-containing moiety on the prime side of the alpha-ketoamide functionality. Like KS-182, this compound also proved to be a fast-binding inhibitor of PfSUB1 but exhibited significantly increased potency, with a calculated *K_i_*_(app)_ of 0.7 μM (Fig. 9E). This enhanced potency was confirmed by dose–response experiments which showed an IC_50_ against PfSUB1 and PkSUB1 of ∼1 μM, and an IC_50_ against PvSUB1 of ∼2 μM (Fig. 9F). Importantly, neither KS-378 nor KS-466 showed any activity against bovine trypsin at concentrations up to 100 μM (data not shown). These results confirm that selective inhibitors based on PfSUB1 substrates can also inhibit both PvSUB1 and PkSUB1, raising the possibility of developing drug-like SUB1-inhibitors that target all three major human malaria pathogens.

## 4 Discussion

We have combined detailed modelling of the SUB1 active site with experimental analysis of substrate specificity and inhibitor sensitivity. Our findings allow us to reach several important conclusions with implications for both improving predictions of SUB1 substrates, and for development of inhibitors with broad potential as antimalarial agents.

We first demonstrated experimentally that rPfSUB1 can be proteolytically converted to a ‘core’ domain that possesses all of the requirements for protease activity and substrate specificity. The nearly 10-fold increase in proteolytic activity following chymotrypsin treatment probably derives primarily from removal of the inhibitory PD, which is a ∼5 nM inhibitor of its cognate enzyme (Jean et al., 2003). Many subtilisin PDs are highly sensitive to proteolysis (Siezen and Leunissen, 1997), so it was no surprise to find that the PfSUB1 PD is relatively susceptible to degradation by chymotrypsin. We have previously reported (Withers-Martinez et al., 2002) that purified rPfSUB1 tends to aggregate and adsorb non-specifically to glass and other surfaces, making it difficult to manipulate. One additional advantage of the chymotrypsin digestion procedure is that the resulting rPfSUB1_cat_ appears homogeneous and fully soluble, lacking the undesirable ‘sticky’ properties of the native recombinant protein. This characteristic is currently being exploited in crystallisation screens aimed at solving its three-dimensional structure.

Molecular modelling of the PfSUB1 active site groove, as well as that of its *P. vivax*, *P. knowlesi* and *P. berghei* orthologues, together with MD analysis, demonstrated several conserved features of the substrate S1–S4 binding pockets. These include the non-polar nature of the S4 pocket, the restricted S2 pocket resulting from a conserved Lys residue (Lys465 in PfSUB1), and the polar nature of the S1 pocket. Contributing to the latter is a free Cys residue (Cys521 in PfSUB1) which is conserved in all of the SUB1 orthologues. The presence of a free Cys close to the active site is not unprecedented in subtilisins; for example, thermitase, proteinase K and cerevisin each have a free Cys close to the catalytic His, which is accessible to mercury compounds. PfSUB1 is potently inhibited by pHMB, and it seems likely that Cys521 is the target of that compound. It is possible that this feature could be exploited in the design of drugs based on active site-directed compounds possessing suitable alkylating ‘warhead’ functions.

A particularly interesting prediction of our modelling analysis was the indication that – uniquely for a subfamily S8A subtilisin as far we are aware – prime side interactions may play important roles in substrate binding by PfSUB1 and its orthologues. The highly basic overall nature of the S′ pocket and the presence of the conserved Lys (Lys465 in PfSUB1), which not only restricts the size of the S2 pocket but may also form stabilizing salt-bridge interactions with P1′, is completely consistent with the clear preference of PfSUB1 for acidic or hydroxyl-containing prime side substrate residues. This was experimentally confirmed by modification of the SERA4st1 substrate; simultaneous substitution with Ala of the P1′ and P3′ acidic residues resulted in a dramatic decrease in its susceptibility to cleavage by PfSUB1. Our evidence that the architecture of the S′ pocket of PvSUB1 and PkSUB1 is identical to that of PfSUB1 implies a similar prime side preference for these enzymes, and an examination of predicted SUB1 processing sites in *P. knowlesi* and *P. vivax* SERA family members is in accord with that prediction (Supplementary Table S2 and Arisue et al., 2011), revealing a similarly strong tendency towards acidic residues, Ser and Thr in the P1′–P4′ positions. This clearly increases confidence that the SERA proteins are indeed substrates for SUB1 in those *Plasmodium* spp., just as in *P. falciparum*. The PbSUB1 active-site architecture is more divergent, and more work is required to elucidate the wider implications of this. However, the differences revealed here between the P1′ preference of PbSUB1 and PfSUB1, together with the observed presence of P1′ Ser in likely important PbSUB1 cleavage sites such as its own putative autocatalytic processing site, and cleavage sites in the essential *P. berghei* proteins PbMSP1 and PbSERA3 (peptides based on some of which were shown here to be cleaved as predicted by rPbSUB1), implies co-evolution of protease and substrates following divergence of these *Plasmodium* spp. from their common ancestor. In the light of our findings here, it may be significant that attempts to replace the *P. berghei pbsub1* gene with the *pfsub1* gene by targeted homologous recombination have consistently failed (Tewari, Yeoh, Billker and Blackman, unpublished data), consistent with the notion that the two orthologues are not functionally interchangeable due to their distinct substrate preferences. It could be imagined that a defect in proteolytic processing of even a single critical *P. berghei* substrate would be sufficient to render transgenic parasites harbouring such an allelic replacement non-viable.

It is important to make an additional point regarding SUB1 substrate specificity. Previous work on processing of both MSP1 and SERA5 by PfSUB1 has shown that cleavage at the various sites within these large molecules takes place in an ordered manner. Thus, in SERA5 cleavage occurs at site 1 before site 2 (Yeoh et al., 2007), whereas in MSP1 cleavage at the most C-terminal PfSUB1 site occurs more slowly than at the other positions (Child et al., 2010). Since SUB1 is a processing protease (rather than purely a degradative enzyme), it is plausible that there may be a biological role for some authentic substrate sequences being cleaved better (i.e. more rapidly) than others. In the light of this, a simple view of substrate preference based on allocating equal ‘weight’ to an assemblage of known target sequences (as in Supplementary Fig. S3) may be misleading, as it does not take into account the likelihood that certain sequences may have evolved to be much better substrates than others. In the light of our findings here regarding the role of prime side interactions, it may therefore be no coincidence that the best PfSUB1 substrate identified to date based on a physiological cleavage site – SERA4st1 – contains exclusively acidic residues at the P1′–P4′ positions. In contrast the slowest-cleaved authentic substrates identified (Child et al., 2010), those based on the 38/42 processing site in two allelic forms of MSP1, are relatively deficient in acidic or hydroxyl-containing prime side residues (their sequences are VVTGE ↓ AISVT and VVTGE ↓ AVTPS). Collectively, our data suggest a rational basis for these differences in substrate preference, and provide information that will be invaluable in searching for new SUB1 target sequences. A valuable tool in understanding the function of SUB1-mediated processing is mutagenesis of established cleavage sites, and our findings also suggest that appropriate substitution of prime-site residues may aid in blocking cleavage.

It is interesting to consider the prime side requirements of SUB1 in the light of its maturation mechanism. In those subfamily S8A and S8B subtilases where proteolytic maturation has been well studied, protease activation follows a single cleavage at the PD-mature domain boundary. This results either in PD degradation, or a second precise cleavage within the PD itself which results in its removal. In the case of subtilisin BPN′, X-ray crystallographic studies have shown that, following cleavage, a large conformational shift occurs in which the segment corresponding to sequence on the prime side of the scissile bond (i.e. the N-terminus of the nascent mature protease domain) moves 25 Å from the active site groove to leave it unobscured (Gallagher et al., 1995; Jain et al., 1998). Activation of furin is a two-step process; initial cleavage takes place at Arg107 but this is not sufficient for furin activation, since PD removal is only achieved following further furin-mediated cleavage at a second site (Arg75) within the PD, following acidification (Anderson et al., 1997, 2002). By contrast to both these mechanisms, in PfSUB1 the second cleavage takes place downstream of the first cleavage – that is, within the C-terminal product of the primary cleavage event (Sajid et al., 2000) – suggesting that the prime side segment may initially remain in the active site groove, blocking access of exogenous substrate to the active site until the second cleavage event takes place. The prime side interactions observed here may aid in stabilising this intermediate complex (Supplementary Fig. S6). Further work is required to explore this model, but it may represent a novel maturation process for subtilisins.

Our evidence that the human malaria pathogen SUB1 orthologues share very similar active site structures led us to explore the potential for producing inhibitors with the capacity to inhibit all three proteases. Peptide-based inhibitors generally have poor bioavailability and pharmacokinetics, but have proven to be excellent starting points for the development of substrate-based inhibitors (e.g. Chen and Njoroge, 2010). Our initial identification here of KS-182 as an inhibitor of PfSUB1, PvSUB1 and PkSUB1 provided proof-of-principle that the similar active-site architecture of these enzymes should allow the development of suitable broad-spectrum non-peptidic inhibitors. Alpha-ketoamides have the important advantage over several other electrophilic pharmacophores that synthetic elaboration on both sides of the alpha-ketoamide function is possible. A good recent example of how this characteristic has been exploited is in the production of selective inhibitors of the Hepatitis C virus (HCV) NS3 protease where, due to the shallow substrate binding groove of this unusual serine protease, it was necessary to develop compounds that make extended interactions on both prime and non-prime sides of the catalytic Ser. Two non-peptidic alpha-ketoamides, Boceprevir and Teleprevir (VX-950), were recently licensed by the FDA for clinical use to treat HCV infection (Kwong et al., 2011; Rizza et al., 2011). These were developed from early 11 residue-long (undecapeptide) peptidyl alpha-ketoamides extending from P6–P5′, demonstrating the power of a substrate-based approach. KS-182 lacks prime side components, so in view of our evidence for the importance of prime side interactions in substrate binding, we produced derivative KS-466 which possesses a prime side carboxyl moiety designed to mimic the acidic residues important for efficient recognition of substrate SERA4st1. The substantially increased potency of this compound compared with KS-182 supports our model. In preliminary experiments, we have found that neither KS-182 nor KS-466 show activity against the parasite at concentrations up to 100 μM, but this is unsurprising as both compounds likely lack good membrane-permeability properties due to their charged nature. Ongoing work is aimed at modifying these compounds to reduce their mass and peptidic nature, whilst enhancing membrane permeability. At present, we have no plausible explanation for the 2-fold higher IC50 values obtained for both KS-182 and KS-466 against PvSUB1 compared with PfSUB1 and PkSUB1, given our evidence that the active-sites of PvSUB1 and PkSUB1 are identical.

What is the potential of SUB1 inhibitors as future antimalarial drugs? There is no obvious human homologue of *Plasmodium* SUB1, the structurally most closely related human enzyme being tripeptidyl-peptidase II (TPP-II; MEROPS ID S08.090), an exopeptidase which releases tripeptides from the N-terminus of peptides, and which has a substrate specificity quite unrelated to that of SUB1 (Rockel et al., 2012). Human lysosomal tripeptidyl-peptidase I (MEROPS ID S53.003) is the only mammalian member of the S53 sedolisin family, so is structurally distinct from SUB1, with a distinct substrate specificity (Tian et al., 2006). Finally, furin and the eight related proprotein convertases belong to the distinct S8B subtilase subfamily; they are therefore also structurally distinct in a number of important ways and exhibit a very different substrate specificity, centred around a dibasic recognition motif at the P2–P1 positions (see Seidah, 2011, for a recent review). We therefore believe that the risk of off-target effects from the in vivo use of appropriately potent SUB1 inhibitors is small. Inhibitors of SUB1 could form a novel and widely applicable addition to the current dwindling armoury of antimalarial agents.

## Appendix A Supplementary data

Supplementary figures

Supplementary figure captions
