# Supplementary material for: Plasmodium subtilisin-like protease 1 (SUB1): Insights into the active-site structure, specificity and function of a pan-malaria drug target
Source: Int J Parasitol. 2012 May 15;42(6):597–612. doi: 10.1016/j.ijpara.2012.04.005 (PMC3378952; doi:10.1016/j.ijpara.2012.04.005)
Supplement: Supplementary materials [file mmc1.pdf]

1. Supplementary Materials and methods

1.1 General

Solvents were purified and dried by standard procedures prior to use; petroleum ether of boiling range 60 – 80 ^{o}C was used. All reactions were performed under an argon atmosphere. Flash chromatography was carried out using Merck Kieselgel (230 – 400 mesh). Thin layer chromatography was performed on silica gel and was visualized by staining with $KMnO_4$. NMR spectra were recorded on Varian Mercury spectrometer (600, 400 and 200 MHz) with chemical shifts values (δ) in ppm relative to TMS as internal standard. LC/MS were performed on Waters 2695 Alliance instrument, column: phenomenex, Gemini 5u C18 11OA, 50x2 mm, 5 µm; mobile phase: acetonitrile –0.1 % aq. HCOOH. Reagents and starting materials were obtained from commercial sources and used as received. (*S*)-3-Amino-*N*-cyclopropyl-2-hydroxypentanamide hydrochloride was prepared according to the literature method (Han et al., 2003). Tert-butyl 3-(3-amino-2-hydroxybutanamido) propanoate was prepared by analogy.

1.2. Synthetic schemes for peptidic ketoamide KS-182, KS-466 and peptidic aminoalcohol KS-378

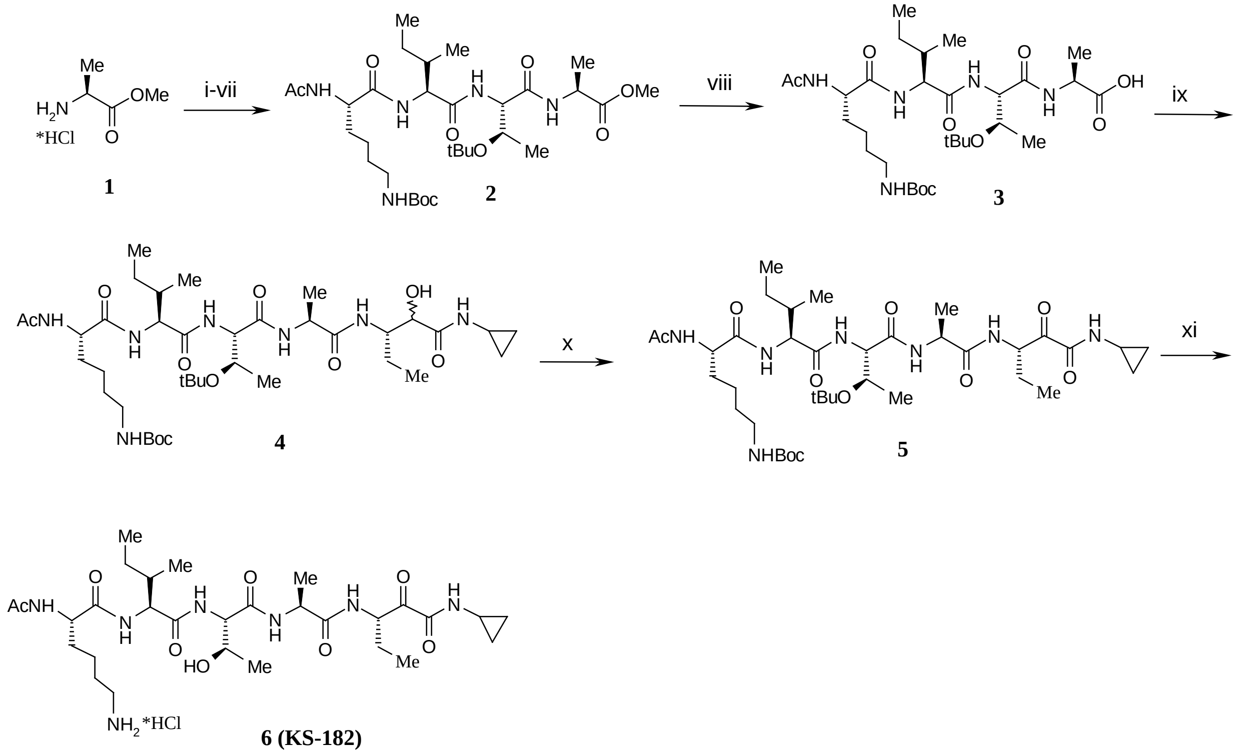

Scheme 1. Reagents and conditions: (i) Fmoc-Thr(tBu)-OH, EDCI, HOBt, DIEA, DCM, r.t., 8 h; (ii) 4-(aminomethyl) piperidine, DCM, r.t., 1 h; (iii) Fmoc-Ile-OH, EDCI, HOBt, DIEA, DCM, r.t., 8 h; (iv) 4-(aminomethyl) piperidine, (v) Fmoc-Lys(Boc)-OH, EDCI, HOBt, DIEA, DCM, (vi) 4-(aminomethyl) piperidine, r.t., 8 h; (vii) acetic anhydride, pyridine, DMF, r.t., 8 h; (viii) LiOH, THF : H_2O (20:1), r.t., 5 h; (ix) (*S*)-3-amino-*N*-cyclopropyl-2-hydroxypentanamide hydrochloride, EDCI, HOBt, DIEA, DCM, r.t., 8 h; (x) Dess-Martin periodinane, NMP, r.t., 23 h, (xi) TFA : DCM (1:1), r.t., 2 h, then HCl in Et_2O.

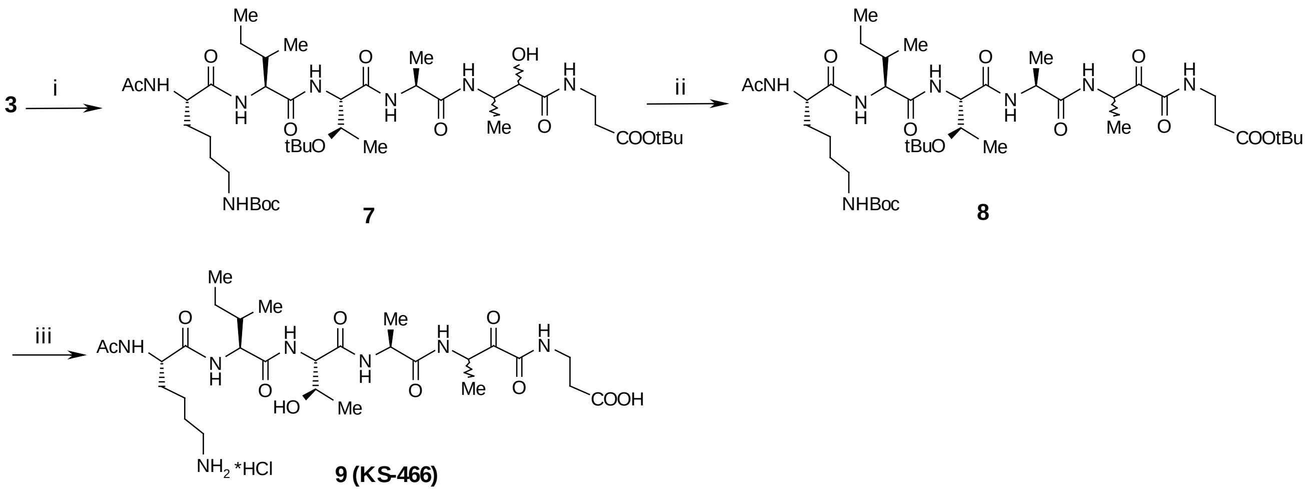

Scheme 2. Reagents and conditions: (i) tert-butyl 3-(3-amino-2-hydroxybutanamido)-propanoate, EDCI, HOBt, DIEA, DCM, r.t., 8 h; (ii) Dess-Martin periodinane, NMP, r.t., 23 h, (iii) TFA : DCM (1:1), r.t., 2 h, then HCl in Et_2O.

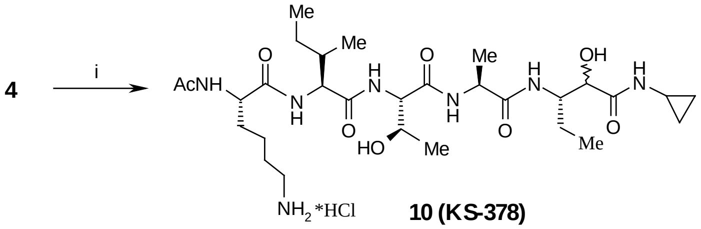

Scheme 3. **Reagents and conditions**: (i) TFA : DCM (1:1), r.t., 2 h, then HCl in Et_2O.

1.3. Description of experiments

Synthesis of (10*S*,13*S*,16*S*,19*S*)-methyl-10-acetamido-16-((*R*)-1-tert-butoxyethyl)-13-sec-butyl-2,2,19-trimethyl-4,11,14,17-tetraoxo-3-oxa-5,12,15,18-tetraazaicosan-20-oate (2)

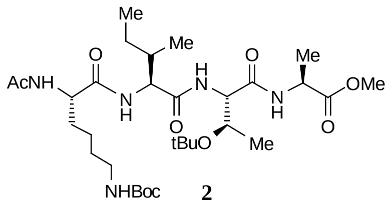
2

A mixture of Fmoc-Thr(tBu)-OH (1.00 g, 2.5 mmol), Ala-OMe*HCl (0.38 g, 2.7 mmol), EDCI (0.43 g, 2.7 mmol) and HOBt (0.37 g, 2.7 mmol) and DIEA (0.88 mL, 5.0 mmol) in DCM (20 ml) was stirred for 8 h at room temperature (TLC control –

hexane : ethyl acetate, 4:1). The reaction mixture was washed with H_2O (5x10 ml) and then with brine (10 ml). Organic phase was dried over Na_2SO_4, filtrated and evaporated in vacuo. The residue was purified by flash chromatography on silica gel eluting with (hexane: ethyl acetate, 4:1) to provide Fmoc-Thr(tBu)-Ala-OMe (0.95 g, 95%) as a colourless crystalline compound. LC/MS: 484 (M+1).

Intermediate (0.95 g, 2.0 mmol) was dissolved in DCM (20 mL) and 4-aminomethyl piperidine (0.89 g, 7.8 mmol) was added to the solution. The resulting mixture was stirred for 1 h at room temperature, during this time the suspension was formed. The mixture was filtered and the solution obtained was washed with 10% (w/v) aqueous phosphate buffer (pH 5.5, 3x5 mL). Organic phase was separated, dried over $Na_2SO_{4,}$ filtered and evaporated in vacuo to obtain crude Thr-Ala-OMe (0.42 g, 82%) as a yellowish solid.

To the solution of Thr(tBu)-Ala-OMe (0.42 g, 1.6 mmol) in DCM (20 mL), Fmoc-Ile-OH (0.50 g, 1.5 mmol), EDCI (0.25 g, 1.6 mmol), HOBt (0.21 g, 1.6 mmol) and DIEA (0.38 mL, 2.1 mmol) were added in the given order and the resulting mixture was stirred for 8 h at room temperature (TLC control- hexane : ethyl acetate, 4:1). The reaction mixture was washed with H_2O (5x10 ml) and then with brine (10 ml). Organic phase was dried over Na_2SO_4, filtrated and evaporated in vacuo. The residue was purified by flash chromatography on silica gel eluting with hexane : ethyl acetate (4:1) to provide Fmoc-Ile-Thr-Ala-OMe (0.52 g, 58%) as a colourless crystalline compound. LC/MS: 597 (M+1).

Intermediate (0.52 g, 0.87 mmol) was dissolved in DCM (20 ml) and 4-aminomethyl piperidine (0.39 g, 3.4 mmol) was added to the solution. The resulting mixture was stirred for 1 h at room temperature, during this time the suspension was formed. The mixture was filtered and the solution obtained was washed with 10% (w/v) aqueous phosphate buffer (pH 5.5, 3x5 mL). Organic phase was separated, dried over Na_2SO_4, filtered and evaporated in vacuo to obtain crude Ile-Thr(tBu)-Ala-OMe (0.31 g, 60 %) as a yellowish solid.

To the solution of Ile-Thr(tBu)-Ala-OMe (0.31 g, 0.83 mmol) in DCM (20 mL), Fmoc-Lys(Boc)-OH (0.22 g, 0.46 mmol) , EDCI (0.080 g, 0.51 mmol), HOBt (0.069 g, 0.51 mmol) and DIEA (0.09 mL, 0.56 mmol) were added in the given order and the resulting mixture was stirred for 8 h at room temperature (TLC control - hexane: ethyl acetate, 4:1). The reaction mixture was washed with H_2O (5x10 ml) and then with brine (10 ml). Organic phase was dried over Na_2SO_4, filtrated and evaporated in vacuo. The residue was purified by flash chromatography on silica gel

eluting with hexane: ethyl acetate, 1:1 to provide Fmoc-Lys(Boc)-Ile-Thr(tBu)-Ala-OMe (0.26 g, 61%) as a colourless crystalline solid. LC/MS: 825 (M+1)

Intermediate (0.26 g, 0.31 mmol) was dissolved in DCM (20 ml) and 4-aminomethyl piperidine (0.14 g, 1.26 mmol) was added to the solution. The resulting mixture was stirred for 1 h at room temperature, during this time the suspension was formed. The mixture was filtered through and the solution obtained was washed with 10% (w/v) aqueous phosphate buffer (pH 5.5, 3x5 mL). Organic phase was separated, dried over Na_2SO_4 filtered and evaporated in vacuo to obtain a crude Lys(Boc)-Ile-Thr(tBu)-Ala-OMe (0.18 g, 96%) as a yellowish solid.

Pyridine (0.026 mL, 0.32 mmol) and Ac_2O (0.031 mL, 0.32 mmol) were added to the solution of Lys(Boc)-Ile-Thr(tBu)-Ala-OMe (0.18 g, 0.30 mmol) in DCM (20 mL) and the mixture stirred for 1 h at room temperature (TLC control- hexane: ethyl acetate, 4:1). The reaction mixture was washed with H_2O (5x10 ml) and then with brine (10 ml). Organic phase was dried over Na_2SO_4, filtrated and evaporated in vacuo. The residue was purified by flash chromatography on silica gel eluting with ethyl acetate to provide with **2** (0.14 g, 73 %) as a colourless crystalline compound.

^{1}H NMR : (400 MHz; DMSO-d_6;TMS) δ: 0.78-0.80 (8H, m); 0.96 (3H, d, 6.0 Hz); 1.07 (9H, s); 1.10-1.40 (18H, m); 1.52 (1H, m); 1.71 (3H; s); 2.81 (2H, m); 3.56(3H, s); 3.98-3.99 (1H, m); 4.19-4.26 (5H, m); 6.68 (1H, m),7.58 (1H, d, 8.8 Hz); 7.84-7.93 ppm (3H, m). LC/MS: 645 (M+1)

Synthesis of (10*S*,13*S*,16*S*,19*S*)-10-acetamido-16-((*R*)-1-tert-butoxyethyl)-13-sec-butyl-2,2, 19-trimethyl-4,11,14,17-tetraoxo-3-oxa-5,12,15,18-tetraazaicosan-20-oic acid (3)

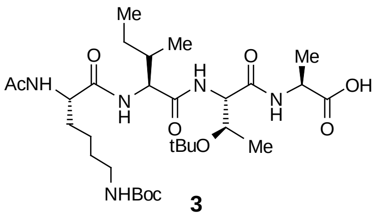

3

A mixture of **2** (0.14 g, 0.21 mmol), LiOH (0.051 g, 2.1 mmol) in THF : H_2O (20:1, 10 mL) was stirred at room temperature for 3 h and then acidified to pH 2 by addition of 1 M HCl. Water (10 mL) was added and the mixture extracted with ethyl acetate (10x3 mL). Organic phase was washed with brine, dried over Na_2SO_4 and evaporated in vacuo to provide compound **3** as a colourless solid (0.12 g, 88 %).

^{1}H NMR: (400 MHz; DMSO-d_6;TMS) δ: 0.76-0.80 (6H, m); 0.95 (3H, dd, 6.4 and 2.4 Hz); 1.07 (9H, s); 1.12-1.23 (1H, m); 1.24 (1H, m); 1.71 (3H; d; 6.8 Hz); 1.32 (9H, s); 1.40-1.50 (1H, m); 1.50-1.60 (1H, m); 1.70-1.77 (2H, m); 1.78 (3H, d, 5.2 Hz),1.95 (1H, s); 2.81 (2H, q) ; 3.13 (1H, d, 4.8Hz); 3.58-3.54 (2H, t, 6.8 Hz); 3.90-3.92 (1H, m); 4.14-4.22 (4H, m); 6.68 (1H, t, 4.6 Hz); 7.58 (1H, d, 8.8 Hz); 7.68-7.73 (1H, m); 7.89-7.94 (1H, m); 8.02 (1H, d, 8.8 Hz); 12.5 ppm (1H, s). LC/MS : 631(M+1); 653 (M+Na).

Synthesis of tert-butyl (3*S*,6*S*,9*S*,12*S*,15*S*)-15-acetamido-9-((*R*)-1-tert-butoxyethyl)-12-sec-butyl-1-(cyclopropylamino)-3-ethyl-2-hydroxy-6-methyl-1,5,8,11,14-pentaoxo-4,7,10,13-tetraazanonadecan-19-ylcarbamate (4)

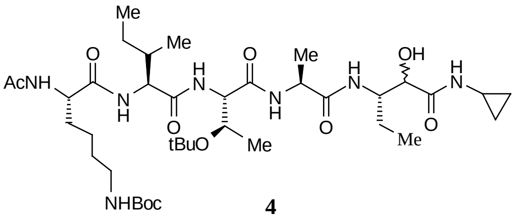

4

A mixture of **3** (50 mg, 0.08 mmol), (*S*)-3-amino-*N*-cyclopropyl-2-hydroxypentanamide hydrochloride (19 mg,0.09 mmol), EDCI (14 mg, 0.09 mmol), HOBt (12 mg, 0.09 mmol), DIEA (20 uL, 0.1 mmol) and DCM (20 mL) was stirred at room temperature for 8 h. Organic phase was washed with brine, dried over Na_2SO_4 and evaporated in vacuo. The residue was purified by column chromatography on silica gel eluting with ethyl acetate followed by a mixture of ethyl acetate and methanol (10 : 1) to provide compound **4** as a yellowish solid (59 mg, 94%).

^{1}H NMR: (400 MHz ; DMSO-d_6 ; TMS), δ: 0.47 -0.50 (2H, m); 0.52-0.61 (2H, m); 0.70-0.90 (10H, m); 0.95-1.10 (3H, d, 6.4 Hz); 1.15-1.30 (14H, m); 1.35 (9H, s); 1.51 (2H, m); 1.75 (1H, m); 1.81 (3H, s); 2.85 (2H, m); 3.78-4.00 (2H, m); 4.20-4.90 (4H, m); 6.71 (1H, t, 6.6 Hz); 7.22 (1H, m); 7.62-7.63 (3H, m) ; 7.95 ppm (2H, m). LC/MS: 784 (M+1), 802 (M+1+H_2O)

Synthesis of tert-butyl (3*S*,6*S*,9*S*,12*S*,15*S*)-15-acetamido-9-((*R*)-1-tert-butoxyethyl)-12-sec-butyl-1-(cyclopropylamino)-3-ethyl-6-methyl-1,2,5,8,11,14-hexaoxo-4,7,10,13-tetra-azanona-decan-19-yl carbamate (5)

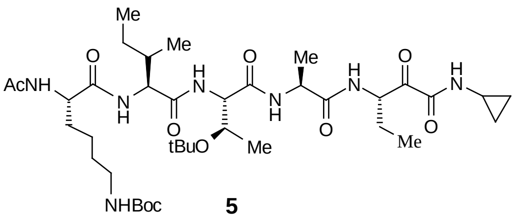

5

A stirred solution of compound **4** (25 mg, 0.02 mmol) in anhydrous NMP (10 ml) was cooled to 0-5°C under N_2 and to this was added DMP (28 mg, 0.06 mmol). The reaction mixture stirred for 23 h at room temperature. Organic phase was washed with $Na_2S_2O_3$ (3x5 mL), $NaHCO_3$ (3x5 mL), H_2O (3x5 mL), dried over Na_2SO_4 and evaporated in vacuo. The residue was purified by column chromatography on silica gel ethyl acetate followed by a mixture of ethyl acetate and methanol (10 : 1) to provide **5** as a yellowish solid (17 mg, 68%).

^{1}H NMR: (400 MHz ; DMSO-d_6 ; TMS), δ: 0.57 -0.66 (2H, m); 0.80-0.90 (8H, m); 0.97-0.99 (3H, d, 6.4 Hz); 1.12-1.24(13H, m); 1.37-1.60 (13H, m); 1.76-1.87 (5H, m); 2.73-2.75 (1H, m); 2.85-2.87 (2H, m); 3.94-3.95 (1H, m); 4.21-4.38 (4H, m); 4.86-4.87 (1H, m); 6.71-6.74 (1H, m); 7.60-7.61 (1H, m); 7.62-7.63 (1H, m); 7.95 (1H, d, 8 Hz); 8.05-8.10 (1H, m); 8.14 (1H, t, 6.6 Hz); 8.72 ppm (1H, d, 5.2 Hz). LC/MS = 783 (M+1), 805 (M+Na), 683 (M+1 - Boc).

Synthesis of (*S*)-2-acetamido-6-amino-*N*-((2*S*,3*S*)-1-((2*S*,3*R*)-1-((*S*)-1-((*S*)-1-(cyclopropyl-amino)-1,2-dioxopentan-3-ylamino)-1-oxopropan-2-ylamino)-3-hydroxy-1-oxobutan-2-yl-amino)-3-methyl-1-oxopentan-2-yl)hexanamide hydrochloride (6) (KS-182).

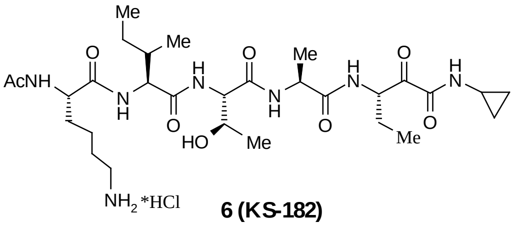

6 (KS-182)

A mixture of **5** (10 mg, 0.01 mmol) in TFA : DCM (1:1, 10 ml) was stirred at room temperature for 2 h. The reaction mixture was concentrated *in vacuo* and treated with HCl (gass) in diethyl ether (3x2 mL, evaporation after each addition). The residue was treated with CH_3CN (3x5 mL, the precipitate was separated by centrifugation after each addition) and then with treated with Et_2O (3x5 mL, the precipitate was separated by centrifugation after each addition). The product was dried over NaOH in vacuo to provide with compound **6** as a yellowish crystalline material (8.1 mg, 95%).

^{1}H NMR: (400 MHz; DMSO-d_6; TMS), δ: 0.56 -1.06 (9H, m); 1.09-1.48 (7H, m); 1.82 (3H, s); 2.11-2.14 (2H, m); 2.73-2.82 (3H, m); 3.95-4.00 (4H, m); 4.21-4.23 (2H, m); 4.88 (1H, m); 7.29 (2H, m) ; 7.70-7.83 (3H, m) ; 8.05-8.08 (1H, m); 8.71-8.76 ppm (1H, m). LC/MS: 626 (M+1)

Synthesis of (10S,13S,16S,19S,22SR,23SR)-tert-butyl 10-acetamido-16-((R)-1-tert-butoxyethyl)-13-sec-butyl-23-hydroxy-2,2,19,22-tetramethyl-4,11,14,17,20,24-hexaoxo-3-oxa-5,12,15,18,21,25-hexaazaoctacosan-28-oate 7

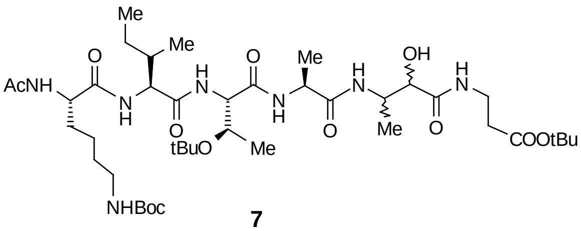

7

A mixture of **3** (100 mg, 0.15 mmol), tert-butyl 3-(3-amino-2-hydroxybutanamido)propanoate (19 mg, 0.18 mmol), EDCI (14 mg, 0.18 mmol), HOBt (12 mg, 0.18 mmol), DIEA (33 uL, 0.18 mmol) and DMF (20 mL) was stirred at room temperature for 8 h. Organic phase was washed with brine, dried over Na_2SO_4 and evaporated in vacuo. The residue was purified by column chromatography on silica gel eluting with ethyl acetate followed by a mixture of ethyl acetate and methanol (10 : 1) to provide compound **7** (48 mg, 35%).

^{1}H NMR: (400 MHz ; DMSO-d_6 ; TMS), δ: 0.70 -0.90 (9H, m); 0.92-1.00 (3H, m); 1.01-1.25 (12H, m); 1.40-1.50 (19H, m); 1.51-1.56 (1H, m); 1.57-1.64 (1H, m); 1.80 (3H, s); 2.40-2.45 (2H, t, 7 Hz); 2.80-2.90 (2H, m); 3.25 (2H, t, 7 Hz); 3.84-3.93 (2H, m); 4.06-4.23 (5H, m); 5.71 (1H, d, 5.6 Hz); 6.68 (1H, t, 5.2 Hz); 7.50-7.76 (4H, m) ; 7.90-7.93(1H, m) ppm. LC/MS: 859 (M+1), 881 (M+Na), 759 (M-Boc+1), 645 (M-Boc-2tBu).

Synthesis of (10S,13S,16S,19S,22SR)-tert-butyl 10-acetamido-16-((R)-1-tert-butoxyethyl)-13-sec-butyl-2,2,19,22-tetramethyl-4,11,14,17,20,23,24-heptaoxo-3-oxa-5,12,15,18,21,25-hexaazaoctacosan-28-oate (8)

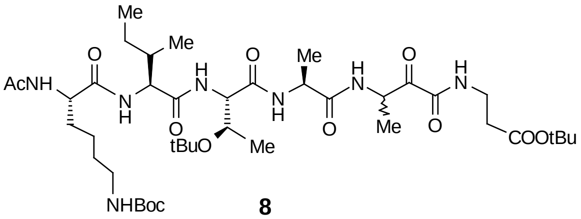

8

A stirred solution of compound **7** (30 mg, 0.03 mmol) in anhydrous NMP (10 ml) was cooled to 0-5°C under N_2 and to this was added DMP (31 mg, 0.06 mmol). The reaction mixture stirred for 23 h at room temperature. Organic phase was washed with $Na_2S_2O_3$ (3x5 mL), $NaHCO_3$ (3x5 mL), H_2O (3x5 mL), dried over Na_2SO_4 and evaporated in vacuo. The residue was purified by column chromatography on silica gel ethyl acetate followed by a mixture of ethyl acetate and methanol (10 : 1) to provide **8** as a yellowish solid (20 mg, 66%).

^{1}H NMR = (400 MHz; DMSO- d_6; TMS) , δ: 0.70-0.85 (6H, m); 1.82 (3H, d); 1.11 (9H, s); 1.12-1.20 (6H, m); 1.22-1.49 (20H, m); 1.50-1.53 (2H, m); 1.60-1.63 (2H, m); 1.80 (3H, s); 1.81-1.95 (2H, m); 2.10 (1H, s); 2.11-2.15 (2H, t, 8 Hz); 2.36-2.40 (2H, t, 7 Hz); 2.65 (3H, s); 2.76-2.82 (2H, m); 3.83-3.98 (1H, m); 4.15-4.31 (5H, m); 4.87-4.96 (1H, d, m); 6.68 (1H, s) ; 7.56-7.58 (1H, d, 7.6 Hz); 7.65-7.81 (1H, m); 7.90-8.03 (2H, m) ; 8.16-8.18 (1H, d, 6.4 Hz); 8.59-8.64 (1H, q, 5.6 and 11.2 Hz) ppm. LC/MS = 857 (M+1), 757 (M+1-Boc)

Synthesis of (4S,7S,10S,13S,16SR)-4-(4-aminobutyl)-7-sec-butyl-10-((R)-1-hydroxyethyl)-13,16-dimethyl-2,5,8,11,14,17,18-heptaoxo-3,6,9,12,15,19-hexaazadocosan-22-oic acid (9) (KS-466)

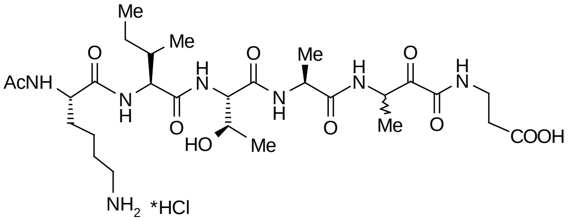

9 (KS-466)

A mixture of **8** (18 mg, 0.021 mmol) in TFA : DCM (1:1, 10 mL) was stirred for 2 h at room temperature and the reaction mixture was concentrated in vacuo. The residue was treated with CH_3CN (3x5 mL, the precipitate was separated by centrifugation after each addition) and then with treated with Et_2O (3x5 mL, the precipitate was separated by centrifugation after each addition). The product was dried over NaOH in vacuo to provide with compound **9** (10 mg, 70 %) as a yellowish crystalline solid.

^{1}H NMR = (400 MHz; DMSO-d_6; TMS), δ: 0.70-0.91 (8H, m); 1.82 (3H, d); 1.00-1.10 (6H, m); 1.11-1.35 (9H, m); 1.40-1.65 (3H, m); 1.70-2.00 (5H, m); 2.15 (2H, t, 7.8 Hz); 2.44 (2H, t, 7 Hz); 2.72-2.79 (4H, m); 3.27-3.39 (5H, m); 4.17-4.29 (4H, m); 4.94 (1H, m); 7.68-7.77 (3H, m); 7.92-8.04 (1H, m); 8.60-8.68 (1H, m) ppm. LC/MS = 644 (M+1), 662 (M+1+H_2O).

Synthesis of (2S)-2-acetamido-6-amino-N-((2S,3S)-1-((2S,3R)-1-((2S)-1-((3S)-1-(cyclo-propylamino)-2-hydroxy-1-oxopentan-3-ylamino)-1-oxopropan-2-ylamino)-3-hydroxy-1-oxobutan-2-ylamino)-3-methyl-1-oxopentan-2-yl)hexanamide hydrochloride 10 (KS-378)

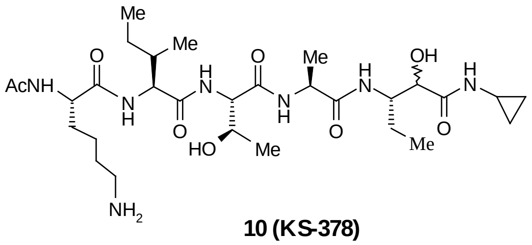

10 (KS-378)

A mixture of **4** (10 mg, 0.012 mmol) in TFA : DCM (1:1, 10 mL) was stirred for 2 h at room temperature and the reaction mixture was concentrated in vacuo. The residue was treated with CH_3CN (3x5 mL, the precipitate was separated by centrifugation after each addition) and then with treated with Et_2O (3x5 mL, the precipitate was separated by centrifugation after each addition). The product was dried over NaOH in vacuo to provide with compound **10** (8 mg, 94.4 %) as a yellowish crystalline solid.

^{1}H NMR: (400 MHz; DMSO-d_6; TMS), δ: 0.40-0.62 (2H, m); 0.70-0.90 (6H, m); 0.95-1.02 (2H, m); 1.10-1.35 (12H, m); 1.40-1.60 (2H, m); 1.81 (3H, s); 2.72 (1H, d, 4.8 Hz); 2.80-3.10 (2H, m); 3.80-4.10 (2H, m); 4.15-4.32 (3H, m); 7.63-7.80 (3H, m); 7.85-8.00 (2H, m); 8.01 ppm (1H, d, 10 Hz). LC/MS: 628 (M+1).

References

Han, W., Hu, Z., Jiang, X., Wasserman, Z.R., Decicco, C.P. 2003. Glycine α-ketoamides as HCV NS3 protease inhibitors. Bioorg Med Chem Lett 13, 1111-1114.
