# Supplementary material for: Plasmodium subtilisin-like protease 1 (SUB1): Insights into the active-site structure, specificity and function of a pan-malaria drug target
Source: Int J Parasitol. 2012 May 15;42(6):597–612. doi: 10.1016/j.ijpara.2012.04.005 (PMC3378952; doi:10.1016/j.ijpara.2012.04.005)
Supplement: Supplementary Tables [file mmc2.doc]

**Supplementary Table S1.** Comparative list of structurally equivalent residues^a^ in two bacterial subtilisins and *Plasmodium* subtilisin-like protease 1 (SUB1) orthologues.

| **Active site** | **Subtilisin** | **Subtilisin** |  |  |  |  |
| --- | --- | --- | --- | --- | --- | --- |
| **Pockets** | **Novo BPN'** | **Carlsberg** | **PfSUB1** | **PvSUB1** | **PkSUB1** | **PbSUB1** |
|  | **1TO2** | **1BH6** |  |  |  |  |
|  |  |  |  |  |  |  |
| **S1** | A152 | A152 | S517 | S461 | S470 | S431 |
|  | G154 | G154 | S519 | S463 | S472 | S433 |
|  | G166 | G166 | S537 | A481 | A490 | S451 |
|  |  |  |  |  |  |  |
|  | S125 | S125 | S490 | S434 | S443 | S404 |
|  | P129 | P129 | D494 | D438 | D447 | D408 |
|  | E156 | S156 | C521 | C465 | C474 | C435 |
|  |  |  |  |  |  |  |
|  |  |  |  |  |  |  |
| **S2** | S33 | T33 | S373 | S317 | S326 | S287 |
|  | N62 | N62 | N426 | N370 | N379 | H340 |
|  | L96 | L96 | L461 | L405 | L414 | L375 |
|  | G100 | G100 | K465 | K409 | K418 | K379 |
|  |  |  |  |  |  |  |
|  |  |  |  |  |  |  |
| **S3** | S101 | S101 | L466 | L410 | L419 | L380 |
|  | G127 | G127 | S492 | S436 | S445 | S406 |
|  |  |  |  |  |  |  |
|  |  |  |  |  |  |  |
| **S4** | G102 | G102 | G467 | G411 | G420 | G381 |
|  | Y104 | Y104 | L469 | L413 | L422 | L383 |
|  | I107 | I107 | M472 | M416 | M425 | I386 |
|  | L126 | L126 | F491 | F435 | F444 | F405 |
|  | G128 | G128 | F493 | F437 | F446 | F407 |
|  | S130 | S130 | E495 | E439 | E448 | E409 |
|  | L135 | L135 | F500 | F444 | F453 | F414 |
|  |  |  |  |  |  |  |
| **Triad** | D32 | D32 | D372 | D316 | D325 | D286 |
|  | H64 | H64 | H428 | H372 | H381 | H342 |
|  | S221 | S221 | S606 | S549 | S558 | S519 |
|  |  |  |  |  |  |  |
| **Ox. hole** | N155 | N155 | N520 | N464 | N473 | N434 |
|  |  |  |  |  |  |  |
| **S'** | G215 | T215 | R600 | R543 | R552 | M513 |
|  | A216 | S216 | K601 | K544 | K553 | E514 |
|  | S218 | N218 | N603 | N546 | N555 | S516 |
|  |  |  |  |  |  |  |
|  | F189 | F189 | F575 | F518 | F527 | F488 |

^a^ Residues contributing to active site pockets S1 (orange), S2 (slate blue), S3 (indigo blue), S4 (pink) and S´ (turquoise) for subtilisin BPN´, subtilisin Carlsberg, PfSUB1, PvSUB1, PkSUB1 and PbSUB1. Catalytic triad residues and oxyanion hole partners (which also belong to the S1 pocket) are shown in light grey. Residues in white font indicate amino acid variations between *Plasmodium vivax* (Pv)SUB1, *Plasmodium knowlesi* (Pk)SUB1, *Plasmodium berghei* (Pb)SUB1 and *Plasmodium falciparum* (Pf)SUB1.

**Supplementary Table S2.** Established and predicted subtilisin-like protease 1 (SUB1) cleavage sites in *Plasmodium* serine rich antigen (SERA) family members. List of known and predicted SUB1 cleavage sites in SERA family members from *Plasmodium falciparum* (Pf), *Plasmodium malariae* (Pm), *Plasmodium ovale* (Po), *Plasmodium vivax* (Pv), *Plasmodium knowlesi* (Pk), *Plasmodium berghei* (Pb), *Plasmodium chabaudi* (Pc) and *Plasmodium yoelii* (Py). Predicted SERA amino acid sequences were obtained from Arisue et al. (2011), and the family members are categorised into groups II, III and IV as defined by those authors. Residues flanking the selected positions are shown from P5 to P5´, with P4 to P1 residues in red. Predicted or known cleavage sites are indicated by red downward-pointing arrow. Group I SERA family members do not possess SUB1 cleavage sites (Yeoh et al., 2007). PkSERA2 (Group IV) is truncated and does not contain SUB1 cleavage sites, whilst site 2 is missing from PySERA2.

| **Group** | **Site 1** | **Site 2** |
| --- | --- | --- |
| **Group II**  **PfSERA7**  **PmSERA9**  **PoSERA6**  **PvSERA11**  **PkSERA5**  **PbSERA4**  **PcSERA4**  **PySERA4** | TFKGE↓DEDLD  PTIAQ↓DEEYS  QTRGD↓DDAEE  PIKAQ↓EEDAN  PIKAQ↓EEDAN  IIKGE↓DDLDE  IIKGE↓DESDE  IIKGE↓DDLDE | YISAQ↓DEPPT  IVSGQ↓TEPES  TLLGQ↓SEEGE  VVTGQ↓TETPT  VVDGQ↓AVVTG  SISGQ↓TDNQI  SISGQ↓TDNQV  SISGQ↓TDNQI |
| **Group III**  **PfSERA6**  **PmSERA8**  **PoSERA5**  **PvSERA10**  **PkSERA4**  **PbSERA3**  **PcSERA3**  **PySERA3** | KVKAQ↓DDFNP  KVMGQ↓DDQDS  KTKAQ↓DEENS  KVKGQ↓DELSP  KVKGQ↓DDLNP  KVTAQ↓SDEDS  KITAQ↓SDEDN  KISGQ↓SDEDT | FVHGQ↓SNESD  IIRGQ↓DDPST  SVYGQ↓DEPTD  QISGQ↓DAAAA  QISGQ↓DEDIT  DVSGQ↓SENHQ  DVSGE↓SETSD  DVSGQ↓SEGNQ |
| **Group IV**  **PfSERA4**  **PfSERA5**  **PmSERA6**  **PmSERA7**  **PoSERA3**  **PoSERA4**  **PvSERA8**  **PvSERA9**  **PkSERA2**  **PkSERA3**  **PbSERA1**  **PbSERA2**  **PcSERA1**  **PcSERA2**  **PySERA1**  **PySERA2** | KITAQ↓DDEES  EIKAE↓TEDDD  EIIAQ↓DDEGS  VTQAA↓DDNTN  KIKGQ↓DDDQS  VTQAA↓DDNTN  KVKGQ↓DDEES  AIPAK↓ASDEE  N/A  KLKGQ↓DDEDS  VVTAQ↓SEDEI  LTIGQ↓SDEDN  LARAQ↓SDDEI  LTTAQ↓SDEDN  LVTAQ↓SEDEI  LAVGQ↓SDEDN | YVYGQ↓DTTPV  IIFGQ↓DTAGS  TVHGQ↓AVEES  VIYGS↓DNSAS  SVYGQ↓ATEQT  TVHGE↓ADNVS  AVQGQ↓DTPQE  VLHGQ↓EVAEA  N/A  TVEGQ↓EGTEQ  IIEGQ↓DEPAS  AIFGQ↓DETVQ  LIQGQ↓DEPAK  AIYGQ↓AEPEA  IIEGQ↓NEPAK  N/A |
|  |  |  |

**Supplementary Table S3.** Correct cleavage by recombinant *Plasmodium berghei* subtilisin-like protease 1 (rPbSUB1) of synthetic decapeptides based on *P. berghei* merozoite surface protein 1 (MSP1) and *P. berghei* serine-rich antigen 3 (SERA3) processing sites

| Peptide name  (10-mer) | Peptide sequence^a^ | Digestion product  identified^b^ | Predicted *m/z* | Measured *m/z* |
| --- | --- | --- | --- | --- |
|  |  |  |  |  |
| PbSERA3st2 | Ac-DVSGQ↓SENHQ | Ac-DVSGQ | 547.2 | 546.52 |
| PbMSP1st1 | Ac-TTSGQ↓SSTEP | SSTEP | 520.2 | 519.49 |
| PbMSP1st2 | Ac-VVTGE↓SEETS | Ac-VVTGE | 545.57 | 546.27 |
| PbMSP1st3 | Ac-TTRAE↓SEEDI | Ac-TTRAE | 619.30 | 618.63 |
|  |  | SEEDI | 592.24 | 591.56 |
|  |  |  |  |  |

^a^ See Section 3.5 of the main paper for the details of predicted *P. berghei* MSP1 and SERA3 processing sites and relevant references. Expected scissile bond is indicated by a downward-pointing arrow.

^b^ Digestion products were fractionated by reversed-phase (RP)-HPLC and identified by electrospray mass spectrometry. Note that in most cases the RP-HPLC column did not retain highly polar cleavage products (which eluted in the column flow-through), allowing identification only of the N-terminal product of cleavage.

**References**

Arisue, N., Kawai, S., Hirai, M., Palacpac, N.M., Jia, M., Kaneko, A., Tanabe, K., Horii, T., 2011. Clues to evolution of the SERA multigene family in 18 *Plasmodium* species. PLoS One 6, e17775.

Yeoh, S., O'Donnell, R.A., Koussis, K., Dluzewski, A.R., Ansell, K.H., Osborne, S.A., Hackett, F., Withers-Martinez, C., Mitchell, G.H., Bannister, L.H., Bryans, J.S., Kettleborough, C.A., Blackman, M.J., 2007. Subcellular discharge of a serine protease mediates release of invasive malaria parasites from host erythrocytes. Cell 131, 1072-1083.
