# Supplementary material for: Plasmodium subtilisin-like protease 1 (SUB1): Insights into the active-site structure, specificity and function of a pan-malaria drug target
Source: Int J Parasitol. 2012 May 15;42(6):597–612. doi: 10.1016/j.ijpara.2012.04.005 (PMC3378952; doi:10.1016/j.ijpara.2012.04.005)

Supplementary Fig. S1.

```
                       Signal sequence
PfSUB1_PFE0370C        MMLNKKVVALCTLTLHLFCIFLCLGKEVRSEENGKIQDDAKKIVSELRFLEKVEDVIEKS 60
PvSUB1_PVX_097935      MVLTRRAALLLCPWVIQLVIKRTLAGDILPNEGKKEKDDVHKIISELRFLQKVETILESS 60
PkSUB1_PKH_102540      MVHTARVALLLFPWVVQLLIHRTFASDIVPNDGK--KDDVQKIISELRFLQKVETILENS 58
PbSUB1_PBANKA_110710   ----MRTVFIYACIISLVLRTIPAHNDLMSKEKENKEKDVHKIIEDLRFLEKVDAILENS 56


PfSUB1_PFE0370C        NIGGNEVDADENSFNPDTEVPIEEIEEIKMRELKDVKEEKNKNDNHNNNNNNISSSSSSS 120
PvSUB1_PVX_097935      NMSVSDVEADANAYNPDRDAPKEELQKLQ-----DQQETPSKEPNNLRN-SPQKR----- 109
PkSUB1_PKH_102540      NMSISDVEADANAYNPDKDAPKEELQKIQ-----GQQEDSEKQPSHLRNKNQQEK----- 108
PbSUB1_PBANKA_110710   NMTIDDVKADADAYNPDEDAPKEELNKIE--------MEKKKAEEEAKNSKKKIL----- 103


PfSUB1_PFE0370C        SNTFGEEKEEVSKKKKKLRLIVSENHATTPSFFQESLLEPDVLSFLESKGNLSNLKNINS 180
PvSUB1_PVX_097935      ----AEKKESPGKNKKSLRLIVSENHATSPSFFEESLLQEDVVSFIQSKGKLSNLKNLKS 165
PkSUB1_PKH_102540      ----VEKKASSIKNKKALRLIVSENHATSPSFFEESLLQDEVMSFIQSKGKLSNLKNLKS 164
PbSUB1_PBANKA_110710   -----ERYLLDEKKKKSLRLIVSENHATSPSFFEESLIQEDFMSFIQSKGEIVNLKNLKS 158


                                                   First processing site
PfSUB1_PFE0370C        MIIELKEDTTDDELISYIKILEEKGALIESDKLVSADNIDISGIKDAIRRGEENIDVNDY 240
PvSUB1_PVX_097935      MIIDLNSDMTDEELAEYISLLERKGALIESDKLVGADDVSLASVKDAVRRGESSVNWGKL 225
PkSUB1_PKH_102540      IIIDLNGDMTDEELAEYINMLEKKGALIESDKLVGADDISIASIKDAVRRGEDRVIWEKL 224
PbSUB1_PBANKA_110710   MIIELNSDMTDKELEAYITLLKKKGAHVESDELVGADSIYVDIIKDAVKRGDTSINFKKM 218


PfSUB1_PFE0370C        KS-MLEVENDAEDYDKMFGMFNESHAATSKRKRHSTNERGYDTFSSPSYKTYSKSDYLYD 299
PvSUB1_PVX_097935      RSTMLEVPSGESPP---------SHAASSG--------------------------SPFD 250
PkSUB1_PKH_102540      HSNMLEQKQEEHEH---------GDSAISDNHVNRGN------------------SSNYD 257
PbSUB1_PBANKA_110710   QSNMLEVEN-----------------------------------------------KTYE 231

                                                Chymotrypsin
                                                                 Model starts F339
PfSUB1_PFE0370C        DDNNNNNYYYSHSSNGHNSSSRNSSSSRSRPGKYHFNDEFRNLQWGLDLSRLDETQELIN 359
PvSUB1_PVX_097935      DDDD---LLSEAALHREEAHLAGSKTTKG----YKFNDEYRNLQWGLDLARLDETQDLIN 303
PkSUB1_PKH_102540      DDDDDDGFLSDVSRF-KETHLVGKEKNKS----YKFNDEYRNLQWGLDLARLDETQDLIK 312
PbSUB1_PBANKA_110710   KLNN-------------NLKKSKNSYKKS-----FFNDEYRNLQWGLDLARLDDAQEMIT 273


PfSUB1_PFE0370C        EHQVMSTRICVIDSGIDYNHPDLKDNIELNLKELHGRKGFDDDNNGIVDDIYGANFVNNS 419
PvSUB1_PVX_097935      ANRVSVTKICVIDSGIDYNHPDLRNNIDVNVKELHGRKGVDDDSNGVVDDVYGANFVNNS 363
PkSUB1_PKH_102540      NNRVSVTKICVIDSGIDYNHPDLRNNIDVNVKELHGRKGVDDDNNGVVDDVYGANFVNNT 372
PbSUB1_PBANKA_110710   TNSVETTKICVIDSGIDYNHPDLKGNIYVNLNELNGKEGIDDDNNGIIDDIYGVNYVNNT 333

                                                                S4
PfSUB1_PFE0370C        GNPMDDNYHGTHVSGIISAIGNNNIGVVGVDVNSKLIICKALDEHKLGRLGDMFKCLDYC 479
PvSUB1_PVX_097935      GDPMDDNYHGTHVSGIISAVGNNGIGIVGVDGHSKLVICKALDQHKLGRLGDMFKCIDYC 423
PkSUB1_PKH_102540      GDPMDDNYHGTHVSGIISAIGNNGIGIVGVDGHSKLIICKALDQHKLGRLGDMFKCIDYC 432
PbSUB1_PBANKA_110710   GDPWDDHNHGSHVSGIISAIGNNSIGVVGVNPSSKLVICKALDDKKLGRLGNIFKCIDYC 393

                                 S1      S4                         S1
PfSUB1_PFE0370C        ISRNAHMINGSFSFDEYSGIFNSSVEYLQRKGILFFVSASNCSHPKSSTPDIRKCDLSIN 539
PvSUB1_PVX_097935      ISRQAHMINGSFSFDEYSNIFNASVEHLRSLGIFFFVSASNCAHDKLSKPDIAKCDLAVN 483
PkSUB1_PKH_102540      ISRKAHMINGSFSFDEYSGIFNASVDYLRTLGILFIVSASNCSHDKHRKPDITKCDLAVN 492
PbSUB1_PBANKA_110710   INKKVNIINGSFSFDEYSTIFSSTIEYLARLGILFVVSSSNCSHPPSSIPDITRCDLSVN 453

                                                           S'
PfSUB1_PFE0370C        AKYPPILSTVYDNVISVANLKKNDNNNHYSLSINSFYSNKYCQLAAPGTNIYSTAPHNSY 599
PvSUB1_PVX_097935      HRYPPILSKTHNNVIAVANLKR-DLDESYSLSVNSFYSNIYCQLAAPGTNIYSTTPMNNY 542
PkSUB1_PKH_102540      FRYPPILSRTHNNVIAVGNLMK-DIDNSYSLSVNSFYSTIYCQLAAPGTNIYSTTPFNSY 551
PbSUB1_PBANKA_110710   SKYPSVLSTQYDNMVVVANLKK-KINGEYDISINSFYSDIYCQVSAPGANIYSTASRGSY 512

                         S'
PfSUB1_PFE0370C        RKLNGTSMAAPHVAAIASLIFSINPDLSYKKVIQILKDSIVYLPSLKNMVAWAGYADINK 659
PvSUB1_PVX_097935      RKLNGTSMASPHVAAIASIVRSINPNLTYLQIVEILRNAIVKLPSLTERVSWGGYVDILR 602
PkSUB1_PKH_102540      RKLNGTSMASPHVAAIASIIRSINPNLSYVEIVEIMKNAIVKLPSLKDKVSWGGYVDILR 611
PbSUB1_PBANKA_110710   MELSGTSMAAPHVAGIASIILSINPDLTYKQVVNILKNSVVKLSSHKNKIAWGGYIDILN 572

                   Model ends N662    Chymotrypsin

PfSUB1_PFE0370C        AVNLAIKSK-KTYINSNISNKWKKKSRYLH 688
PvSUB1_PVX_097935      AVNLAIDSKAAPYIKSHSWFRWKQGSRR-- 630
PkSUB1_PKH_102540      AVNLAIDSKEAPYIKSQSWFRWKKRVR--- 638
PbSUB1_PBANKA_110710   AVKNAISSK-NSYIRFQGIRMWKSKKRN-- 599
```

Supplementary Fig. S2.

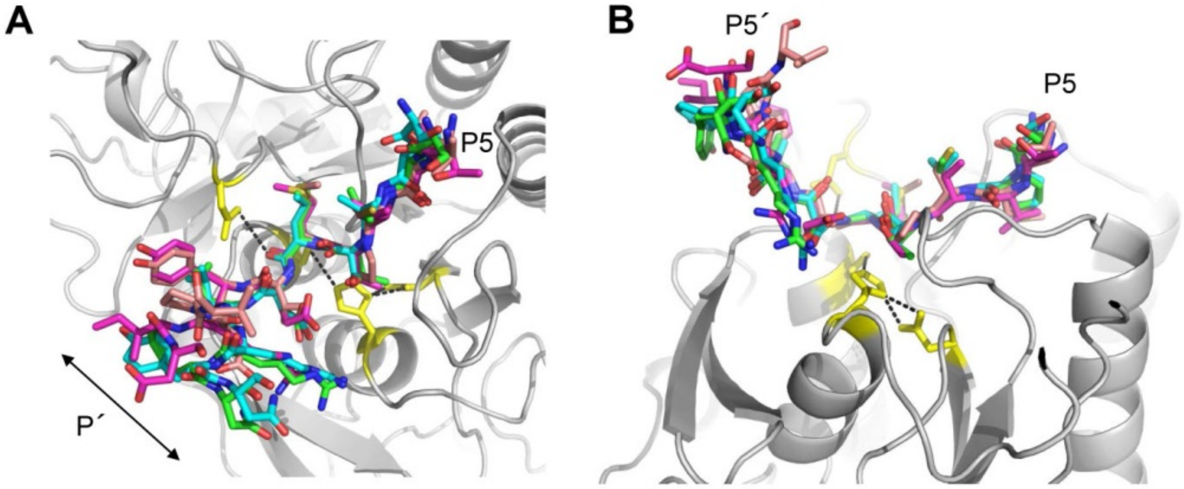

Supplementary Fig. S3.

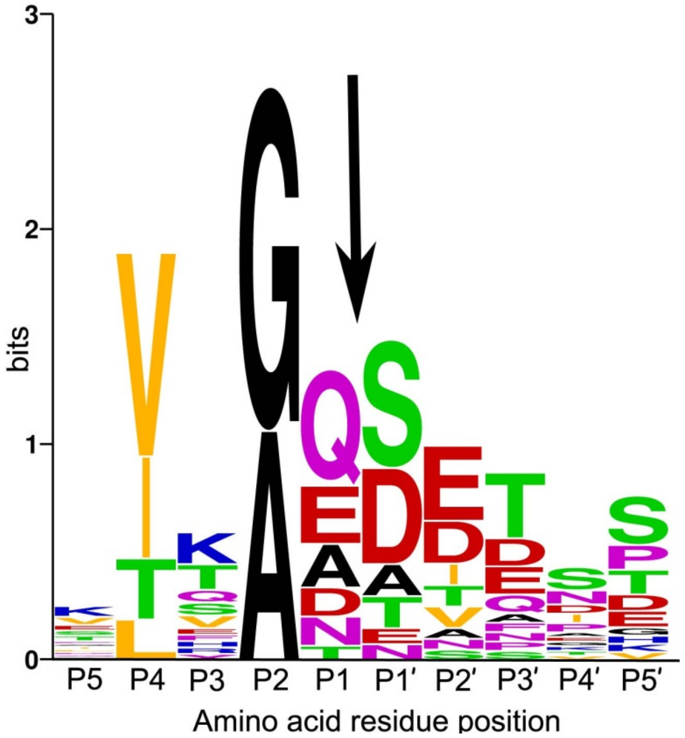

Supplementary Fig. S4.

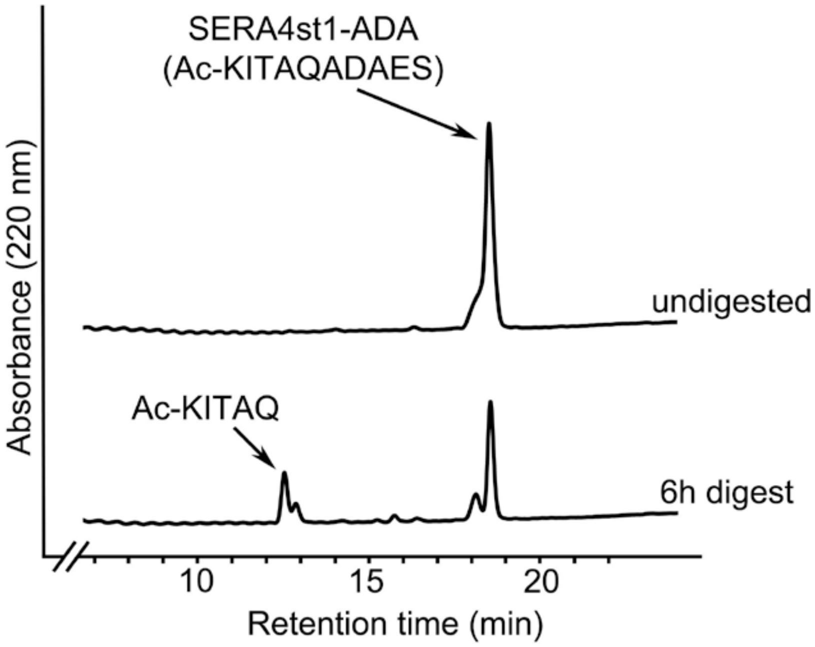

Supplementary Fig. S5.

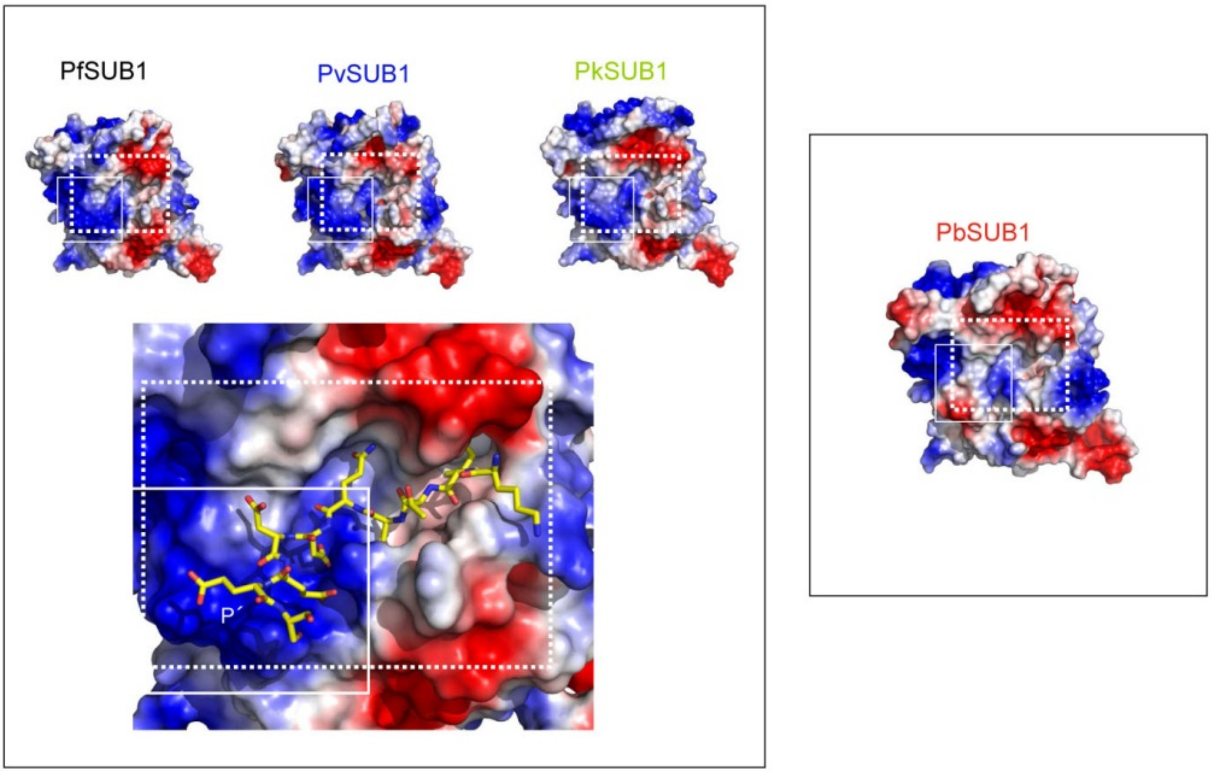

Supplementary Fig. S6.

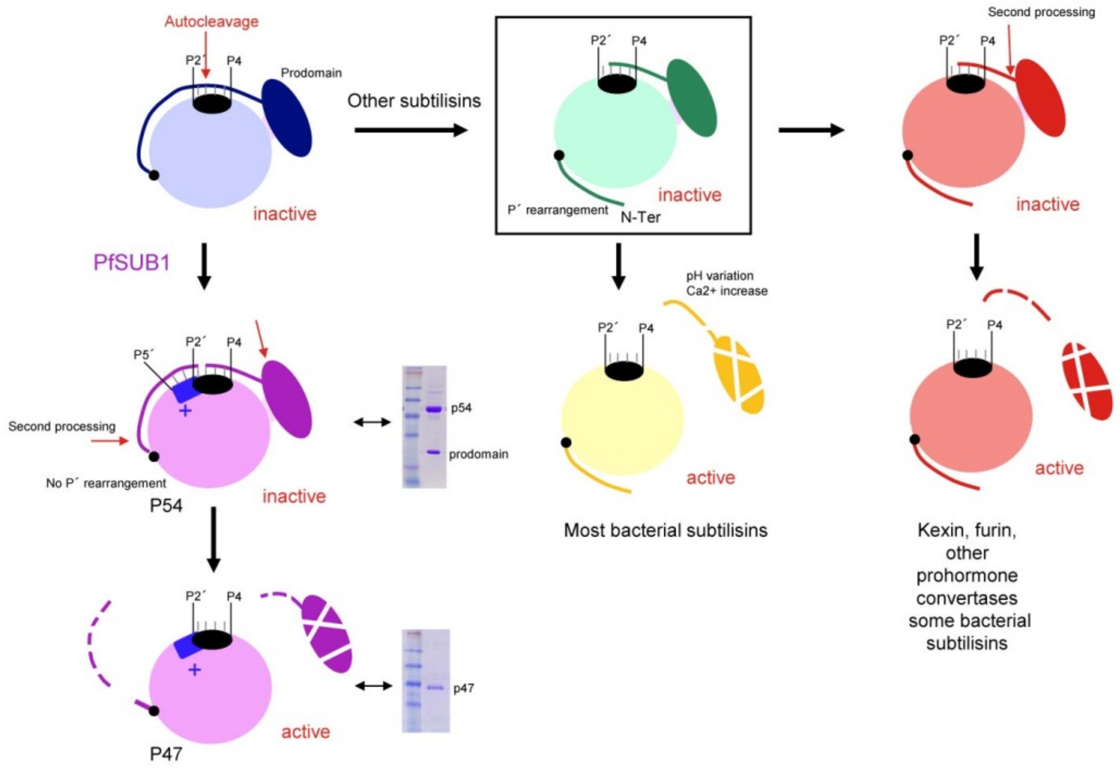

Supplement: Supplementary figures [file mmc3.pdf]
