# Supplementary material for: Plasmodium subtilisin-like protease 1 (SUB1): Insights into the active-site structure, specificity and function of a pan-malaria drug target
Source: Int J Parasitol. 2012 May 15;42(6):597–612. doi: 10.1016/j.ijpara.2012.04.005 (PMC3378952; doi:10.1016/j.ijpara.2012.04.005)
Supplement: Supplementary figure captions [file mmc4.doc]

Supplementary figure legends

Fig. S1. Multiple alignment of the *Plasmodium* subtilisin-like protease 1 (SUB1) sequences examined in this study. Primary sequences of *Plasmodium falciparum* (Pf)SUB1 (PFE0370c), *Plasmodium vivax* (Pv)SUB1 (PVX_097935), *Plasmodium knowlesi* (Pk)SUB1 (PKH_102540) and *Plasmodium berghei* (Pb)SUB1 (PBANKA_110710) were all obtained from PlasmoDB (v8.1, http://www.plasmodb.org/plasmo/home.jsp) and aligned using ClustalW. (v2.0.12). Predicted N-terminal secretory signal peptides are shown in red, as is the processing site at which autocatalytic cleavage takes place to remove the prodomain. Indicated in pale and dark blue is the PfSUB1 segment obtained following limited proteolytic cleavage of recombinant PfSUB1 (rPfSUB1) in vitro with chymotrypsin, whilst the sequence encompassed by the homology model is shown in dark blue. Catalytic triad and oxyanion hole residues are highlighted in red, whilst key residues of the various substrate-binding pockets are shown in yellow (S1), blue (S2), purple (S4) and pale blue (S′). Conserved Cys residues involved in disulphide bond formation, and gaps inserted to maintain alignment, are highlighted in grey.

Fig. S2. Subtilisin-inhibitor structures used for modelling *Plasmodium falciparum* subtilisin-like protease 1 (PfSUB1)-peptide binding. Superimposition of the PfSUB1 model with four X-ray structures of the inhibited bacterial subtilisins BPN′ and Carlsberg. PfSUB1 is represented as a grey cartoon and the bacterial subtilisins have been omitted from the figure for simplicity. The PfSUB1 catalytic residues and oxyanion hole partner side chains are shown as yellow sticks and the hydrogen bond pattern as a dotted line. The protein inhibitors SSI, *Streptomyces* subtilisin inhibitor (PDB ID 2SIC, light pink sticks), eglin C (1CSE and 1SIB, blue and green sticks), and chymotrypsin inhibitor 2 (2SNI, dark pink sticks) have been truncated to a P5–P5′ decapeptide that sits in the enzyme active site. (A and B) Different views of the complex, from which it is clear that the P5–P5′ inhibitor fragments superimpose well, allowing them to be used as templates for modelling the PfSUB1 peptides. Little spatial variation is seen in the P positions where canonical backbone interactions are present, whereas on the P′ side the lack of interactions results in larger variations in conformation of the P3′–P5′ segment as indicated by the arrowed line. In B, the lack of enzyme interaction with the P′ positions is evident, with the P1′–P5′ segment protruding abruptly from the active site.

Fig. S3. Substrate preference of *Plasmodium falciparum* subtilisin-like protease 1 (PfSUB1) based on known authentic cleavage sites. Graphical representation in single-letter code of a multiple sequence alignment of amino acid residues flanking known PfSUB1 cleavage sites within serine-rich antigen 5 (SERA5) (sites 1, 2 and 3), SERA4 and SERA6 (sites 1 and 2), merozoite surface protein 1 (MSP1), MSP6, MSP7, rhoptry-associated protein 1 (RAP1) and MSRP2 (a total of 19 different sequences), plus the internal PfSUB1 autocatalytic processing site at which cleavage occurs during protease maturation. The total height of each residue stack indicates the degree of sequence conservation at that position, while the height of each residue within the stack indicates the relative frequency of that residue at that position. Residues are mostly colour-coded according to the chemical nature of their side-chains (red, acidic (D, E); blue, basic (K, R, H); orange, aliphatic (L, V, I); black, small (G, A); green, uncharged polar with hydroxyl-containing side-chain (S, T); purple, other residues (Q, N, F, P)). The scissile bond is indicated by a black arrow. Residue numbering is according to the system of Schechter and Berger (1967). The figure was produced using the WebLogo facility at http://weblogo.berkeley.edu/logo.cgi and annotated using Adobe Photoshop.

Fig. S4. Prolonged digestion of peptide SERA4st1-ADA with recombinant *Plasmodium falciparum* subtilisin-like protease 1 (rPfSUB1) results in correct cleavage. As shown in Fig. 6 of the text, peptide SERA4st1-ADA (Ac-KITAQADAES) is a very poor substrate for PfSUB1 compared with the parental peptide SERA4st1 (Ac-KITAQ ↓ DDEES; cleavage site depicted by the downward-pointing arrow). In order to confirm that SERA4st1-ADA can be correctly cleaved by PfSUB1, a solution of SERA4st1-ADA (~2 mM in digestion buffer) was incubated for a prolonged period (6 h) at 37 °C in the presence of a high concentration of PfSUB1_cat_ (10 U/ml). Shown are reversed phase-HPLC profiles of the undigested and partially digested peptide after chromatography on a Vydac 4.6 mm × 25 cm C_18_ reversed-phase-HPLC column, eluting with a 4.5–18% (v/v) gradient of acetonitrile in 0.1% TFA over 25 min. The major cleavage product Ac-KITAQ, as identified by electrospray mass spectrometry, is indicated. The major predicted C-terminal cleavage product NH_2_-ADAES was not detected, and would be expected to elute in the column flow-through due to its overall hydrophilic nature. The identity of the additional cleavage digestion product eluting just after the Ac-KITAQ peak was not determined, but could correspond to cleavage at another bond as a result of the prolonged digestion.

Fig. S5. Electrostatic molecular surface potential of the subtilisin-like protease 1 (SUB1) models. PyMOL charge-smoothed protein contact potential representation of *Plasmodium falciparum* SUB1 (PfSUB1), *Plasmodium vivax* SUB1 (PvSUB1), *Plasmodium knowlesi* SUB1 (PkSUB1), *Plasmodium berghei* SUB1 (PbSUB1). The dashed white frame delimitates each enzyme active site whilst the smaller window defines the predicted interaction surface of the substrate P′ residues. The zoomed view shows the modelled binding mode of SERA4st1 (KITAQ ↓ DDEES) in the PfSUB1 active site groove. Prime side acidic substrate residues (DDEE) are stabilized via complementary electrostatic interactions with the positively charged S′ pocket residues. PbSUB1 (right) exhibits less basic electrostatics in the S′ region, indicating likely differences in the P′ side.

Fig. S6. Proposed model for maturation of *Plasmodium falciparum* subtilisin-like protease 1 (PfSUB1) and comparison with bacterial and eukaryotic subfamily S8A and S8B subtilisins. In bacterial subfamily S8A subtilisins, the first autocatalytic processing is immediately followed by a rearrangement of the P′ side of the enzyme, now the new N-terminus of the catalytic domain of the enzyme. The prodomain remains initially associated with the catalytic enzyme, with its C-terminal segments filling the S1–S4 pockets through canonical backbone binding, acting as a competitive inhibitor to prevent access of exogenous substrate. Removal of the cognate prodomain results from its further degradation by the protease itself. In furin, release of the prodomain is triggered by a second precise processing step involving another cleavage within the prodomain. In PfSUB1, we suggest that following the first processing, there is no rearrangement of the P′ side of the molecule due to additional direct stabilizing interactions involving P1′–P3′ positions within the S′ enzyme basic pocket. Complementary charge interactions are also present up to the P5′ residue. At this stage there is no accessibility to the active site on either P or P′ sides. A second processing within the catalytic domain then releases the P′ fragment together with the prodomain and as a result activates the enzyme. If confirmed, this model of activation would be unique in the subtilisin S8A subfamily.
